# High-throughput analysis of N-glycans using AutoTip *via* glycoprotein immobilization

**DOI:** 10.1038/s41598-017-10487-8

**Published:** 2017-08-31

**Authors:** Shuang Yang, David Clark, Yang Liu, Shuwei Li, Hui Zhang

**Affiliations:** 1 Department of Pathology, Johns Hopkins Medicine, Baltimore, MD USA; 2 Institute for Bioscience and Biotechnology Research, University of Maryland College Park, Rockville, MD USA

## Abstract

Analysis of a large number of samples requires an efficient, rapid and reproducible method. Automation is an ideal approach for high-throughput sample preparation. Multi-plexing sample preparation *via* a 96-well plate format becomes popular in recent years; however, those methods lack specificity and require several cleanup steps *via* chromatography purification. To overcome these drawbacks, a chemoenzymatic method has been developed utilizing protein conjugation on solid-phase. Previously, sample preparation was successfully performed in a snap-cap spin-column (SCSC) format. However, sample preparation using SCSC is time-consuming and lacks reproducibility. In this work, we integrated the chemoenzymatic technique in a pipette tip (AutoTip) that was operated by an automated liquid handler. We established a multi-step protocol involving protein immobilization, sialic acid modification, and N-glycan release. We first optimized our automated protocol using bovine fetuin as a standard glycoprotein, and then assessed the reproducibility of the AutoTip using isobaric tags for relative N-linked glycan quantification. We then applied this methodology to profile N-glycans from 58 prostate cancer patient urine samples, revealing increased sialyation on urinary N-glycans derived from prostate cancer patients. Our results indicated AutoTip has applications for high-throughput sample preparation for studying the N-linked glycans.

## Introduction

Glycosylation is one of the most abundant post-translational modifications (PTM) of proteins. By definition, glycosylation in particular refers to the enzymatic attachment of glycans to proteins or lipids^1^. Alteration of glycan structure on the protein can dramatically impact its function, including protein binding, activation, and other biological properties^2^. Therefore, abnormal glycosylation, including aberrant glycan profiles and glycosite occupancy, has been associated with many diseases^3, 4^. Change in protein glycosylation can be monitored for disease occurrence and progression, and be utilized for the identification of specific targets for therapeutic intervention^4–6^.

Protein glycosylation can be analyzed by studying glycopeptides^7, 8^ and glycans^9, 10^ separately, or a combination of both *via* intact glycopeptides analysis^11–14^. Glycopeptides can be enriched by lectin affinity^15, 16^ or chemical immobilization *via* hydrazide-chemistry^17^, termed as solid-phase extraction of glycopeptides (SPEG)^18^. The latter is a chemoenzymatic method that oxidizes the cis-diol of intact glycoproteins or glycopeptides, creating hydrazide-reactive aldehydes for chemical conjugation. To facilitate mass spectrometry analysis, the conjugated N-linked glycoproteins/glycopeptides are then released using the enzyme PNGase F. However, analysis of glycans is distinct from their protein counterparts, with a higher degree of complexity due to a non-template biosynthesis of glycans. High-throughput (HTP) sample preparation has been developed for glycan release, cleanup, and derivatization using different analytical platforms^19^. For example, a polyvinylidene fluoride (PVDF) membrane and in-gel block method has been utilized for HTP N-glycan sample preparation. The released N-glycans are then derivatized by 2-aminobenzamide (2-AB) prior to microplate purification^20^. The entire procedure, including the in-gel block preparation and glycan release, spent a total of three days. To expedite sample preparation, the method can be significantly reduced to 3.5 h by using the rapid deglycosylation kit and instant 2-AB kit from Prozyme^21^. Another cost-effective HTP platform developed incorporated denaturation, reduction, and deglycosylation using a hydrophobic Immobilon-P PVDF membrane filter plate, fluorescent labeling, and 96-well clean-up^22^. More recently, the labeling reagents, RapiFluor, improved sensitivity of detection with chromatographic and MS methodologies for HTP sample preparation of N-glycans^23, 24^.

Recently, a solid-phase capture method, named glycoprotein immobilization for glycan extraction (GIG), has shown potential for applications in a HTP sample preparation for the analysis of N-linked glycans^25–27^. The technique utilizes aldehyde-functionalized resin for conjugation of N-termini or lysine residues of proteins or peptides. Upon immobilization, proteins and glycans can be chemically or enzymatically modified, enabling the analysis of glycans^28^, peptides^29^, and intact glycopeptides^30^. Previously, GIG has been adapted to be integrated in a microfluidic chip format for separation and identification of glycans, enabling robust analysis of even small biological sample amounts^31^. However, the microfluidics chip has the limited capability for processing a large number of samples. For example, the single-channel design limits HTP analysis of multiple sample simultaneously, and would require the addition of more channels. This fact could complicate the design of the microfluidics chip, and also impact reproducibility, which is critical for sample preparation.

Chemoenzymatic-based methods can be integrated in automated sample preparation platforms for rapid and robust analysis of protein glycosylation. To study glycosites from global proteins, a platform based on SPEG technique was adapted into a HTP format using tip-based format^32^. The methodology utilized a pipette tip packed with hydrazide resin for immobilization of glycoproteins after oxidation. Using an automated liquid handler, up to 96 samples can be processed simultaneously, involving glycoprotein conjugation onto the resin, washing, and subsequent enzymatic digestion: first using trypsin to release non-glycosylated peptides, followed by PNGase F digestion to release the glycosite-containing N-glycopeptides. SPEG is highly specific for analysis of glycosite-containing peptides, but glycans are destroyed during oxidation, resulting in loss of glycan component. Despite the advantages of highly specific SPEG approach, workflow based on similar principles that enables the analysis of N-glycans could be further improved using a chemoenzymatic method.

In this study, we designed a novel platform that takes advantage of a pipette tip format (AutoTip), in which aldehyde-functionalized resin is packed, and sample processing is performed by an automated liquid handler. To secure resin in the AutoTip, the resin was sandwiched between the porous filters with pore size is less than 45 μm, which is comparatively much smaller than that of resin (~130 μm). Once anchored in the AutoTip, the resin can be utilized for protein immobilization, glycan modification, and chemoenzymatic digestion. The AutoTip can be operated by an automated liquid handler, processing up to 96 samples simultaneously. The parameters for optimal sample processing were first determined using bovine fetuin as a standard glycoprotein, including duration for protein immobilization, sialic acid modification/derivatization, and N-glycan release. The AutoTip methodology was then applied to N-linked glycan analysis of prostate cancer patient urine sample, demonstrating its applicability to N-glycan analysis of glycoproteins from complex biological samples.

## Results and Discussion

### Coupling time and PNGase F release in SCSC

**Figure 1:**
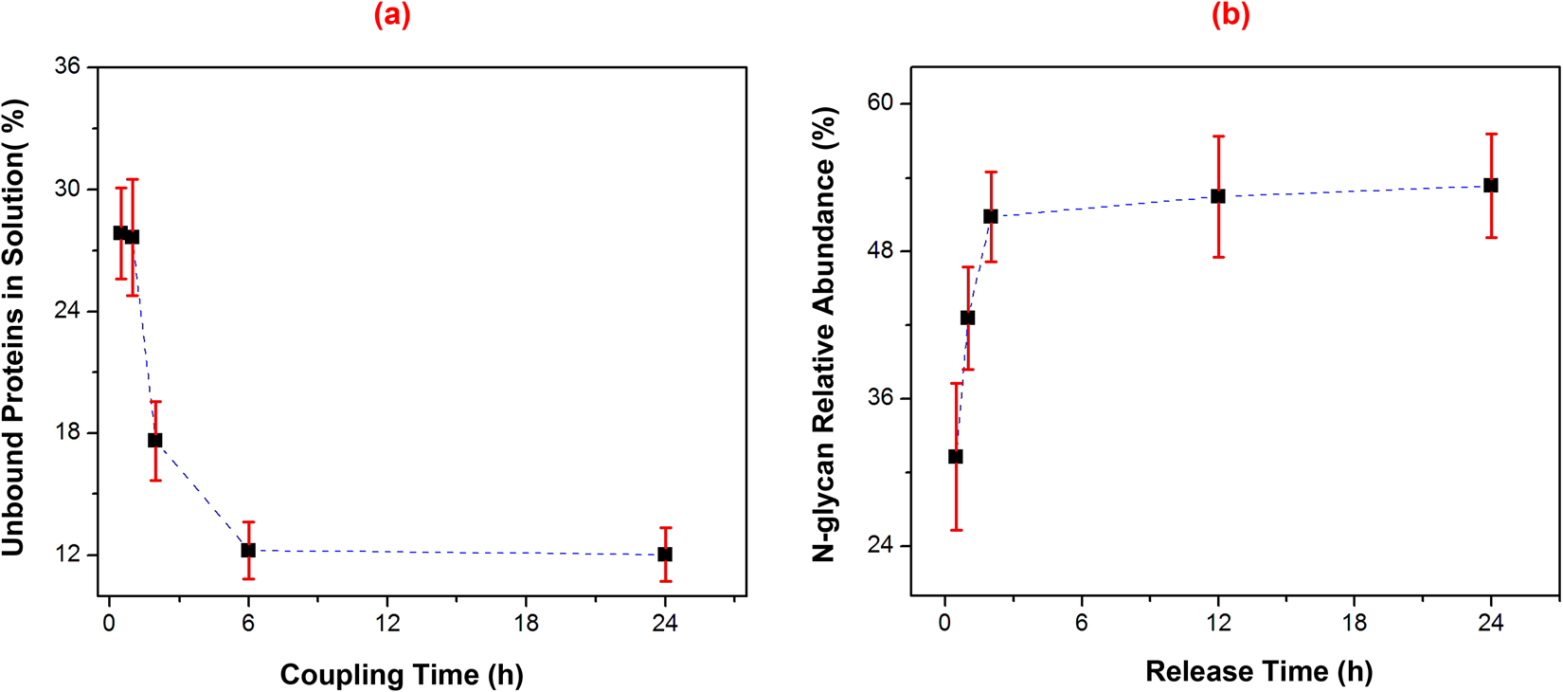
Rate of glycoprotein immobilization and PNGase F release of N-glycans. (**a**) Fetuin was conjugated to Aminolink resin via reductive amination, whereas the unbound proteins in supernatant were measured by a BCA assay. Fetuin was quickly immobilized to the resin within 4 h; (**b**) N-glycans were released by PNGase F in 25 mM ammonium bicarbonate at 37 °C. The PNGase F digestion was complete within 2 h. Four measurement were made for each condition and error bar represented standard deviation.

To compare coupling time and PNGase F release between manual method using a SCSC and automated approach using AutoTip, we first tested protein conjugation and N-glycan release in a SCSC using fetuin. Assuming the total amount of fetuin equals the amount of bound proteins on resin and that of unbound proteins remaining in buffer, we show in Fig. 1a a decreasing amount of unbound protein in solution during the conjugation of fetuin to the resin. Over 85% fetuin was immobilized to the solid support after 4 h, and over 88% after 6 h, via reductive amination. Further, we investigated N-glycan release from the solid-support using PNGase F. An internal standard DP7 (Maltoheptaose; Sigma; 1 µM/1 µL) was added to the N-glycans for quantification^33^. The intensity of N-glycans was normalized to that of DP7. As shown in Fig. 1b, the release of N-glycans (only 1% of N-glycan eluate was used for testing) is saturated after 2 h incubation at 37 °C, indicating complete digestion of N-glycans was achieved.

### Resin capacity for protein immobilization

**Figure 2:**
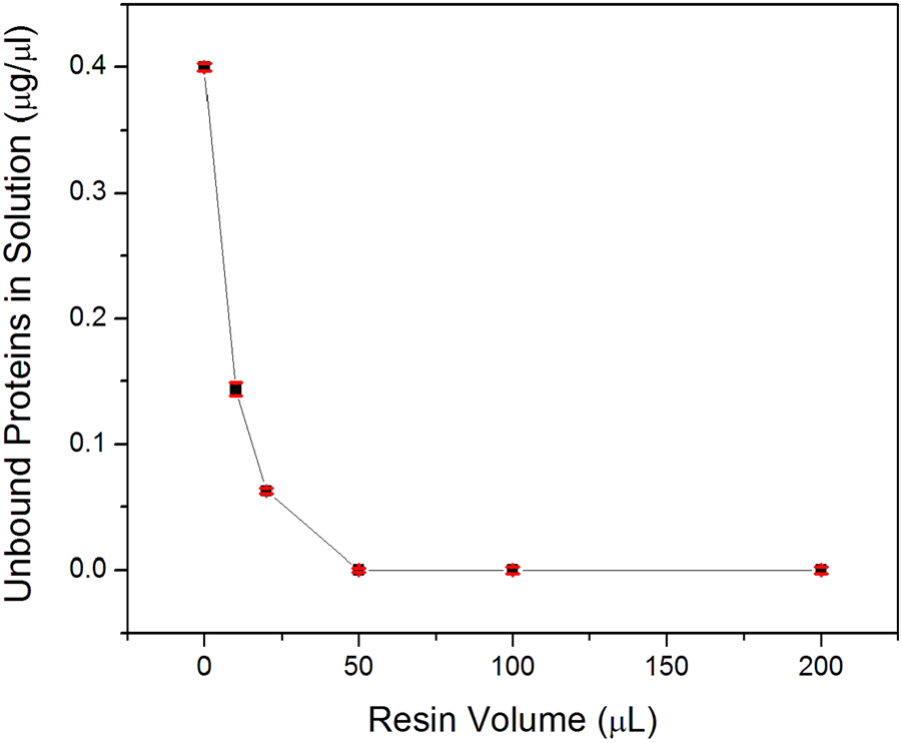
Capacity of Aminolink resin on protein immobilization. Fetuin (200 μg) was added to different volume of Aminolink resin (0, 10, 20, 50, 100, and 200 μl) to the final concentration of 0.4 μg/μl. The conjugation was proceed for 3 hr with mixing in snap-cap spin-column (SCSC). The supernatant was collected for measurement of unbound protein concentration by a BCA assay. Less than 50 μl resin can completely immobilize 200 μg fetuin. Four measurement were made for each condition and error bar represented standard deviation.

To determine the capacity of Aminolink resin for protein immobilization, fetuin (200 μg) was added to different volumes of resin (0, 10, 20, 50, 100, and 200 μl) in a SCSC after pre-conditioning the resin using pH 10 binding buffer (500 μl). After incubation for 3 h at room temperature, the supernatant was collected for measurement of unbound fetuin by a BCA assay. Experiments were conducted in triplicates. Figure 2 shows the concentration of the unbound proteins in binding buffer using different volumes of resin. The initial concentration was 0.4 μg/μl (200 μg in 500 μl binding buffer). More than 60% fetuin was immobilized on 10 μl resin, and over 85% fetuin is conjugated on 20 μl resin. Fetuin (200 μg) was conjugated completely when applied to 50 μl resin material. This result indicated that up to 1 mL of resin material can immobilize at least 4 mg fetuin protein. Although we showed 50 μL of resin material was sufficient for complete protein binding, the volume capacity of the tip was 200 μl. Therefore, we packed 200 μl resin in AutoTip in all experiments.

### AutoTip protein immobilization

**Figure 3:**
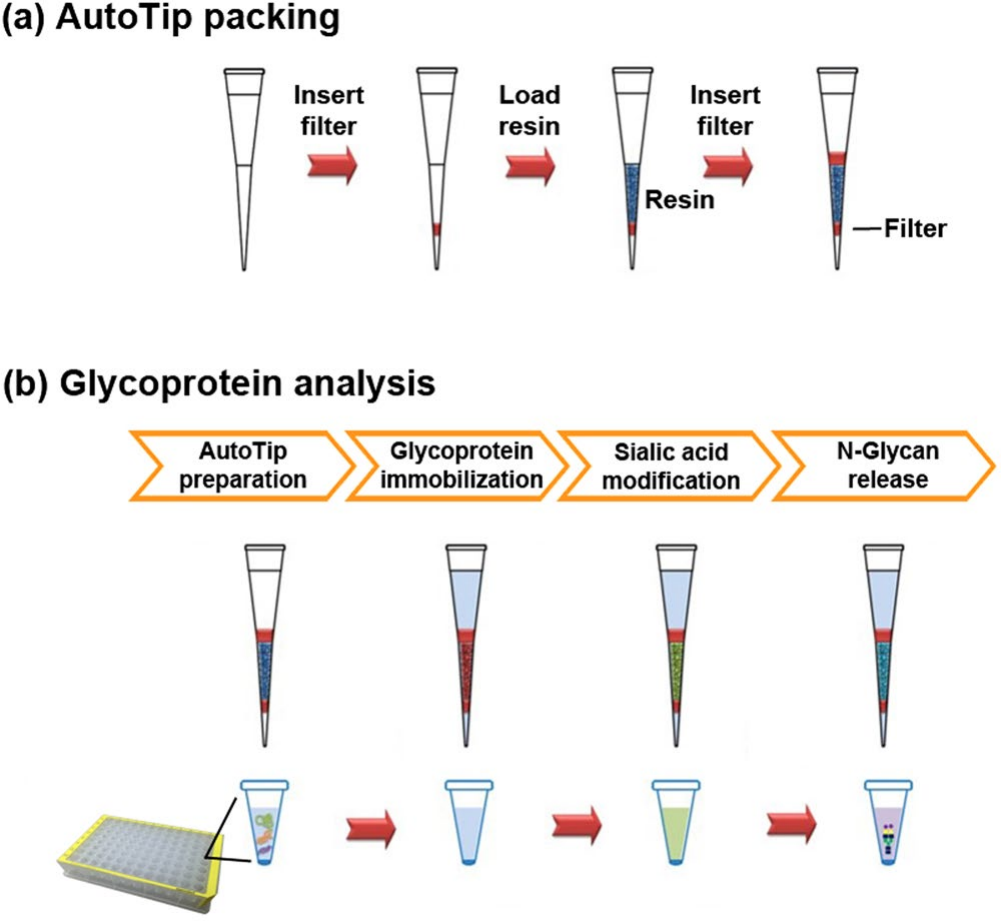
Schematic workflow for packing and analysis of N-glycan using AutoTip. The AutoTip uses pipette tips with capacity from 20–300 μL. (**a**) A polyethylene sheet was inserted into the AutoTip, followed by loading Aminolink resin (200 μL) before sealing AutoTip by another polyethylene sheet. The AutoTip is operated by an automatic liquid handler (e.g., Thermo Versette); (**b**) Glycan analysis: immobilization of protein to AutoTip, modification of glycan, and release of N-glycans. 96-well plate is used for sample preparation.

**Figure 4:**
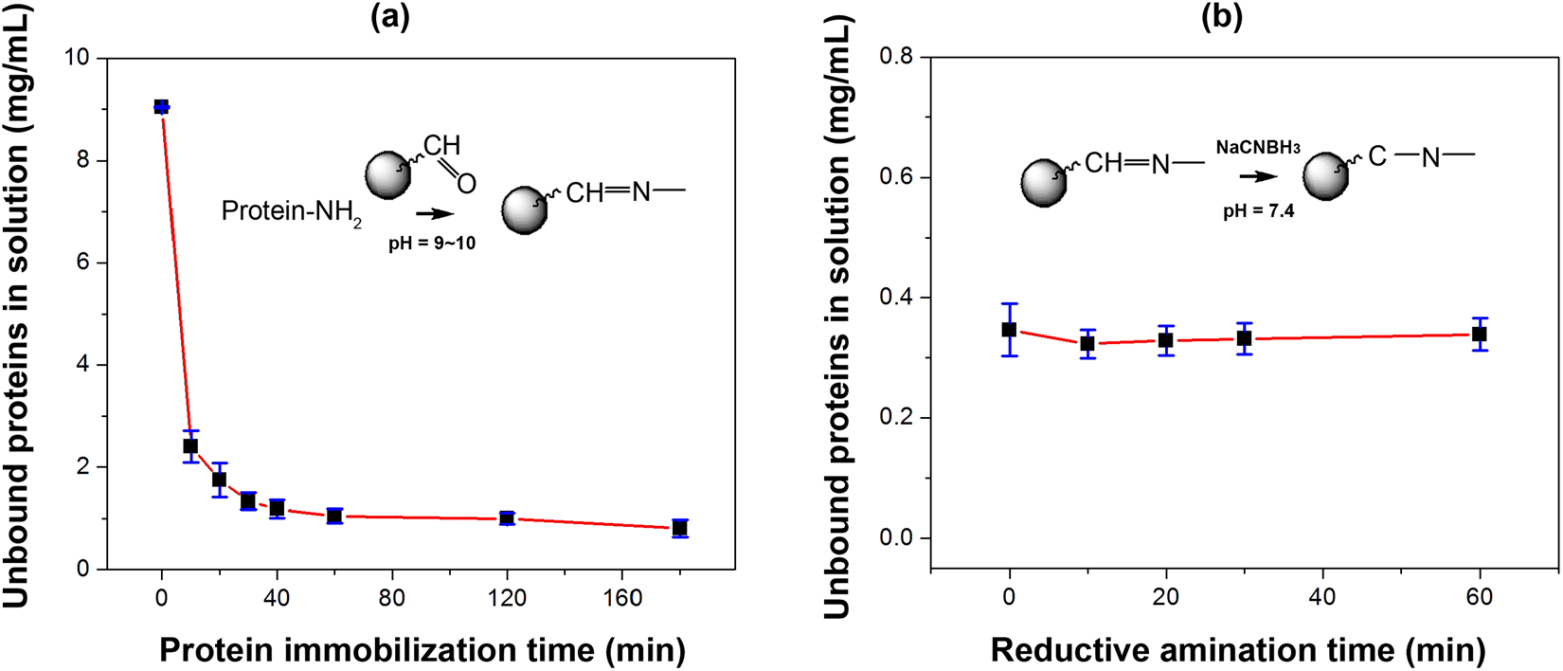
Conjugation of proteins on Aminolink-packed AutoTip. The protein solution was dissolved in sodium citrate and sodium carbonate buffer (pH 9~10). N-termini or lysines were reacted with aldehydes immobilized on the solid support, forming imine that were further reduced to imide. (**a**) The intermediate imine was initiated by nucleophilic addition at mildly basic condition. The unbound proteins were reduced to less than 5% after 20 cycle (each cycle consists of aspiration and dispense of samples) within 40 min; (**b**) The reductive amination was conducted in PBS (pH = 7.4) in the presence of NaCNBH3 (sodium cyanoborohydride). The reaction is approximately accomplished within 30 min (based on the results from four AutoTips).

The conjugation of protein on the resin material is the most critical step for reliable analysis of N-glycans. Using an automated liquid handler, samples were aspirated and dispensed through the Aminolink resin in the AutoTip (Fig. 3). To assess coupling efficiency of the AutoTip, we measured unbound proteins *via* a BCA assay. As shown in Fig. 4a, protein conjugation onto the aldehyde-functionalized resin, leading to the formation of an intermediate imine, was evaluated over a time course. We found over 88% of protein material was immobilized onto the resin after 40 min of incubation (equivalent to ~20 cycles). In contrast, it took over 2–3 h to reach similar coupling efficiency using snap-cap spin column (SCSC) (Fig. 1), possibly due to repeated liquid aspiration and dispensing through the AutoTip, resulting in a thorough interaction between the Aminolink resin aldehyde groups and protein amines. To stabilize the imine bonds, the immobilized proteins were further reacted by reductive amination (Fig. 4b). The reductive amination reaction proceeds at a much faster rate at a lower pH (or 1x PBS, 7.4)^34^, thus PBS buffer was used in the presence of 50 mM NaCNBH_3_. We found the reaction was completed in approximately 20 min using the AutoTip format, allowing the now stably immobilized proteins to be processed in additional chemoenzymatic reactions.

### Derivatization of sialic acids in AutoTip

**Figure 5:**
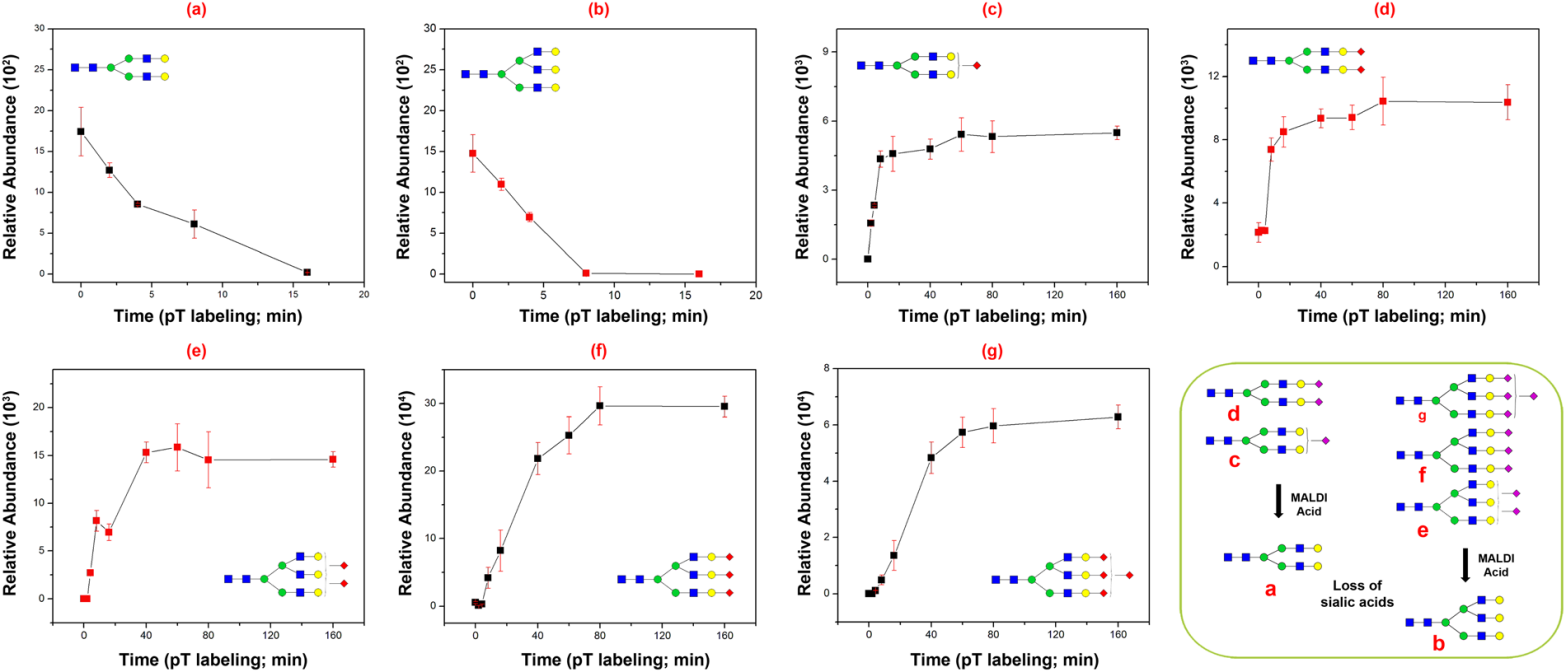
Modification of sialic acids using carbodiimide coupling on AutoTip. Fetuin protein was modified by p-Toluidine in the presence of EDC after immobilization on the beads (Four AutoTips). Sialylated N-glycan, S2H5N4, was used to monitor completion of p-Toluidine-EDC reaction. Without derivatization, MALDI-MS detected no sialic acid H5N4 (**a**) and two sialic acids S2H5N4 (**c**); For incomplete derivatization, MALDI-MS detected (**a**), (**c**), and p-Toluidine labeled two sialic acids S2H5N4 (**b**); When completely labeled, only (**b**) was detected by MALDI-MS.

Sialic acid is a common terminal structure on glycans; however, several factors can result in loss of detectable sialylated residues including: low pH and/or high temperature^35–38^, the composition of organic solvent which strongly impacts signal intensity of sialylated glycans in the negative modes^39^, as well as loss of the sialic acid moiety during MALDI (matrix-assisted laser desorption/ionization) analysis^40^. To stabilize and protect sialic acid residues, we developed a solid-phase chemoenzymatic method for derivatization of sialic acid moiety using carbodiimide coupling^25, 41^. Even though same chemistry is utilized for modification of sialic acids on Aminolink resin, we found that AutoTip format presents an improved efficiency on sialic acid modification, e.g., different linkages of sialic acids are equally derivatized (Supplementary Figure S1). Again, this may be due to the fact that the reagents have a higher surface contact when aspirated and dispensed through the porous beads. Using the SCSC format, complete derivatization of sialic acids with continuously shaking would take over 3 h. In contrast, the liquid handler aspirates and dispenses reagents smoothly through AutoTip every 2 minutes. Thus, the reaction is accomplished within 80 min. To illustrate this, 200 μg of fetuin was coupled to four AutoTips (Note: only 0.1% of released glycans were used for testing) and five sialic acid-containing structures from bovine fetuin (S1H5N4, S2H5N4, S2H6N5, S3H6N5, and S4H6N5) was monitored during p-Toluidine (pT) reaction (Fig. 5c–g). For example, when completely labeled by pT, the mass of S2H5N4 is 2424.0 Da (Fig. 5d) and 1663.7 Da when both sialic acids are lost (Fig. 5a). In our MALDI analysis, the abundance of each glycoform was quantified by comparing the glycan ion intensity to DP7. The ionization efficiency of DP7 could be different from H5N4, S2H5N4 with and without QUANTITY (Quaternary Amine Containing Isobaric Tag for Glycan); however, it is still valid for generating semi-quantification on the relative abundance of molecules present in the sample. With prolonged pT reaction, more S2H5N4 was modified (**d**); the amount of labeled S2H5N4 was increased since the modified sialic acids were stable during MALDI ionization. In contrast, the asialo-glycoform (H5N4 or H6N5 (**b**)) was decreased with the prolonged pT reaction because less unmodified sialylglycoform remained on the resin. The native (or unmodified sialylated glycoform) was minimal during pT reaction, due to loss of the sialic acid residues during MALDI ionization. Recent results revealed that pT or other reaction can completely derivatized α2,3 and α2,6 sialic acids^42, 43^. These results indicated that the reaction was successful and the pT-labeled glycans were stable.

### PNGase F release of N-glycans

Similar to the immobilization and pT derivatization steps, the release of N-glycans by PNGase F was more efficient using AutoTip format because the liquid handler facilitates contact of the PNGase F enzyme with the immobilized glycoproteins. We tested PNGase F digestion dynamics *via* 1) monitoring incubation periods for complete release of N-glycans, and 2) the rate of release for different bovine fetuin N-glycan species. We tested a set of AutoTips using bovine fetuin (20 μg). The immobilized fetuin, after pT derivatization, was released by PNGase F digestion at 1 cycle (2 min), 5 cycles (10 min), 10 cycles (20 min), 30 cycles (60 min), 60 cycles (120 min), and 120 cycles (240 min). Experiments were conducted in triplicates.

Our results indicated release of N-glycans is accomplished within 1 h of PNGase F digestion for majority of N-glycans. We then measured the relative abundance of each Fetuin N-glycan species after PNGase F digestion. Supplementary Figure S2 lists four fetuin N-glycans that have been released at different time points during PNGase F digestion, with their relative abundance determined by the isobaric QUANTITY tag reporter ions (Supplementary Table S1). Several observations are obvious: 1) Release of most N-glycans was accomplished within 1 h digestion. Additional N-glycans can be released with prolonged digestion, but the amount is negligible; 2) N-glycans were effectively released within one cycle of PNGase F digestion. Each cycle takes approximately 2 min, including aspiration, dwelling, and dispensing. Three N-glycans (Supplementary Figure S2a–c), S1H5N4, S2H5N4 and S2N6H5, were detected by MS (MALDI or ESI) after 2 min digestion; 3) The rate of PNGase F digestion was affected by the type of N-glycan. The N-glycan of larger molecular weight, such as S4H6N5 (Supplementary Figure S2d), was detected after 5 cycles (10 min) of PNGase F digestion; 4) Complex N-glycans such as S3H6N5 or S4H6N5 require extended incubation, as we observed slower release of these N-glycans by PNGase. Therefore, a longer period of PNGase F digestion may be more applicable when analysis of complex biological specimens is conducted. This latter observation has been recently reported elsewhere as well^44^. In summary, the release of N-glycans by PNGase F (1 unit per µg glycoprotein) can be accomplished within 1~4 h digestion. It is expected that reaction time can be further reduced if digestion is performed at a higher temperature (37 °C or up to 50 °C) or a larger amount of the PNGase F enzyme is used.

### Reproducibility of AutoTip

To demonstrate the reproducibility of AutoTips, we packed 12 AutoTips for analysis of bovine fetuin N-glycans. Each AutoTip conjugated 20 µg of fetuin after denaturation (88 µL fetuin protein in HPLC water +10 µL denaturing buffer (NEB); 100 °C/10 min). N-glycans were released after sialic acid modification by PNGase F at room temperature (2 h), and subjected to desalting using Hypercarb AutoTips. Twelve N-glycan eluates from the 12 AutoTips were randomly divided into three groups, in which each group was tagged by 4-plex QUANTITY reagents (reporter ion mass: 176, 177, 178, and 179). The labeled N-glycans were re-suspended in 100 µL HPLC water (0.2% formic acid), with a 10 µL aliquot analyzed *via* LC-MS (Supplementary Figure S3).

Table 1 summarized the reproducibility of five released fetuin N-glycans glycan composition, cartoon structure, retention time (RT), and CV (coefficient of variation). (Note: complete results include intensity for each N-glycan from fetuin in Supplementary Table S2; three replicate results are shown in Table S2_run1, S2_run2 and S2_run3). The CV was calculated by comparing ion intensity from QUANTITY reporter ions, and average RT and CV were based on the three AutoTip sets (Table 1). We observed different RTs for glycans containing the same composition. For example, S1H5N4 has two distinct RTs, 16.5 min and 25.3 min respectively; S3H6N5 was detected at three different RTs, 31.5 min, 76.3 min, and 108.7 min, consistent with the reported isomers identified by other methods^45^. The relative abundance of each isomers is also in agreement with the literature, whether quantification was performed by fluorescent labeling^45, 46^ or nuclear magnetic resonance (NMR)^45, 47, 48^. The CV for all AutoTips is below 20%, indicating an excellent reproducibility of AutoTips for glycan analysis.

**Table 1:** Reproducibility of AutoTip on analysis N-glycans from Bovine serum.

Composition	Structure	Isomer	AutoTip Set									Mean		
			1			2			3					
			RT (min)	Intensity	CV (%)	RT (min)	Intensity	CV (%)	RT (min)	Intensity	CV (%)	RT (min)	CV (%)	Intensity
S1H5N4		A	17	2404	16.8	16.3	2104	7.7	16.08	1209	11.8	16.5	12.1	1906
		B	25.8	49401	7.3	25.17	26239	7	24.94	38961	6.8	25.3	7	38200
S2H5N4		A	25.8	83909	8.9	25.23	17578	11.2	25.01	55815	11.4	25.4	10.5	52434
S2H6N5		A	25.4	31505	11.8	24.58	28297	13.5	24.64	31891	10.9	24.9	12.1	30564
S3H6N5		A	32.5	193155	10.7	30.51	159417	9.4	31.34	140108	11.1	31.5	10.4	164227
		B	67.9	12742	15.2	80.76	1305	18.6	80.3	1136	16.3	76.3	16.7	5061
		C	109.6	1404	20.8	10.7.27	3461	15.3	107.79	3141	17.1	108.7	17.7	2669
S4H6N5		A	48.2	21628	19.4	44.85	8292	16.8	45.53	13784	18.4	46.2	18.2	14568
		B	64.6	31540	18	61.33	9396	17.7	60.27	8926	15.9	62.1	17.2	16621

N-glycans were released from AutoTips using an automated liquid handler. The reproducibility was calculated by quantitative analysis of N-glycans from four independent AutoTip by 4-plex QUANTITY tags (176, 177, 178, and 179). The labeled N-glycans were measured by LC-ESI-MS/MS (Thermo Orbitrap Velos). Experiments were performed in triplicates. CV (coefficient of variation) was average of the triplicated tests.

### HTP analysis of N-glycans in urine samples

**Figure 6:**
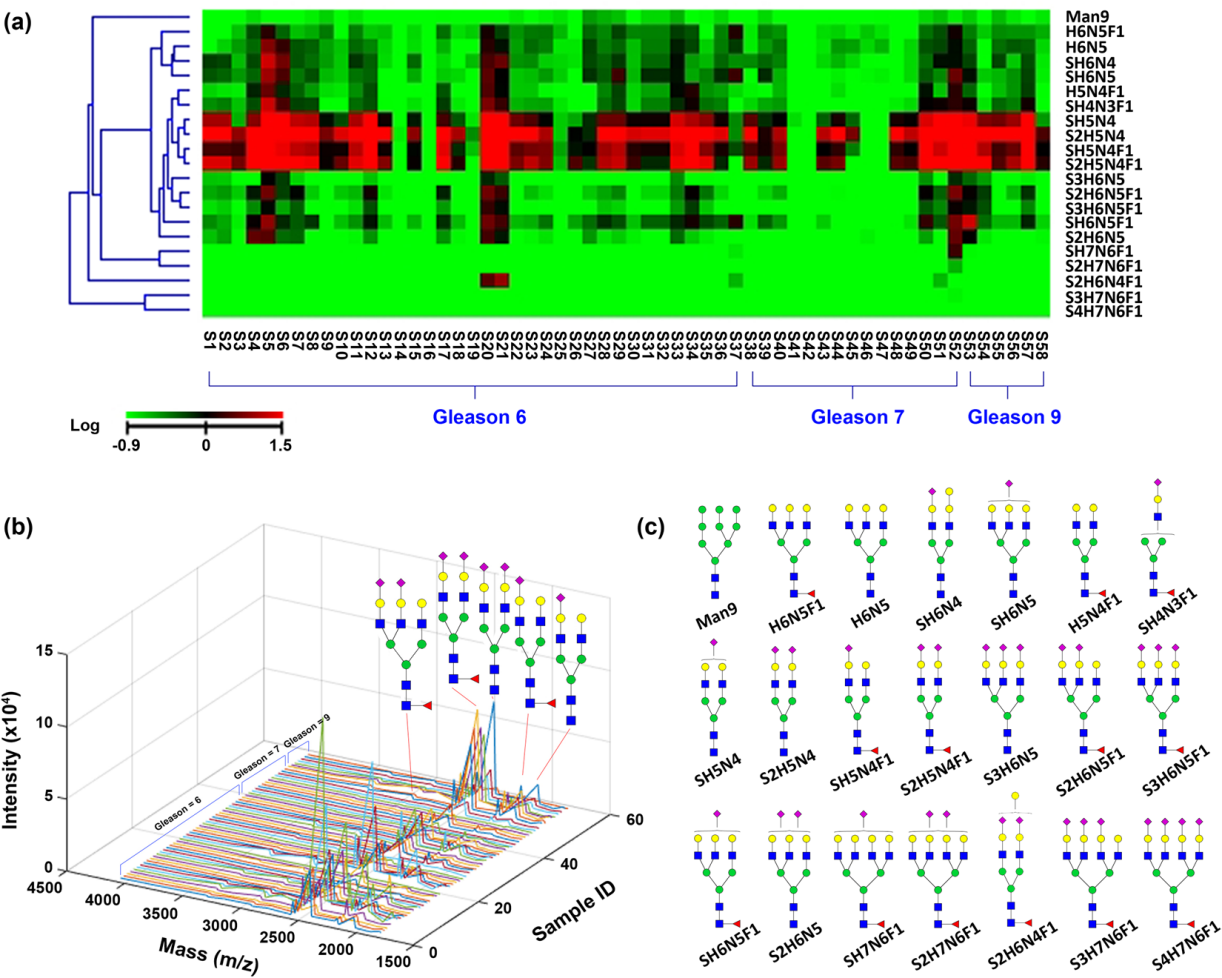
N-glycan profile of prostate cancer urines by MALDI-MS. (**a**) Heat map of 21 N-glycans from 58 prostate cancer urines. Three groups were characterized by AutoTip using a liquid handler, including 38 Gleason 6, 15 Gleason 7, and 6 Gleason 9. The abundance of N-glycans were normalized using mean value of all N-glycans, and heatmap was plotted after being logarithmized. (**b**) Pseudo-3D plot of N-glycans of 58 prostate cancer urines. The sialylated N-glycans were highly abundant in urines. (**c**) Cartoon structures representing the likely isoform of 22 N-glycan compositions identified in urines.

We have collected 58 urine samples from prostate cancer patients, consisting of 37 (Gleason 6), 15 (Gleason 7), and 6 (Gleason 9) patients (Supplementary Table S3 and Figure S4). After removal of salt using centriprep centrifugal filters (10 kDa cut-off), we measured protein concentration of the urine by BCA assay and used 500 µg for N-glycan analysis by AutoTip. The N-glycans were semi-quantitatively analyzed by Shimadzu Maxima Resonance (area under spectra was used for comparison) (Supplementary Table S4). The area under three isotope spectra was summed up for counting abundance of each glycan. To plot a heatmap, we used the average intensity of all N-glycans for normalization. The normalized value was plotted in logarithm for generating the heatmap (Fig. 6a) and the pseudo-3D plot (Fig. 6b).

The results of the urine N-glycome analysis indicated a similar N-glycan profile for prostate cancer patients with Gleason scores 6–9. As shown in Fig. 6a, four out of 22 N-glycans species were highly abundant in almost all 58 samples, specifically the bi-antennary sialylated N-glycans, including S1H5N4, S2H5N4, S1H5N4F1, and S2H5N4F1. Tri-antennary N-glycans were also observed in most prostate cancer patient urine samples, displaying minimal changes across Gleason 6 to 9 scores. The 3D plot indicates that bi-antennary sialylated N-glycans with and without core-fucose are dominant species in prostate cancer patient urine samples. Searching a human glycan database, we identified 22-N-glycan species identified in our analysis of prostate cancer patient urine samples, of which seventeen N-glycans were sialylated (Fig. 6c).

**Figure 7:**
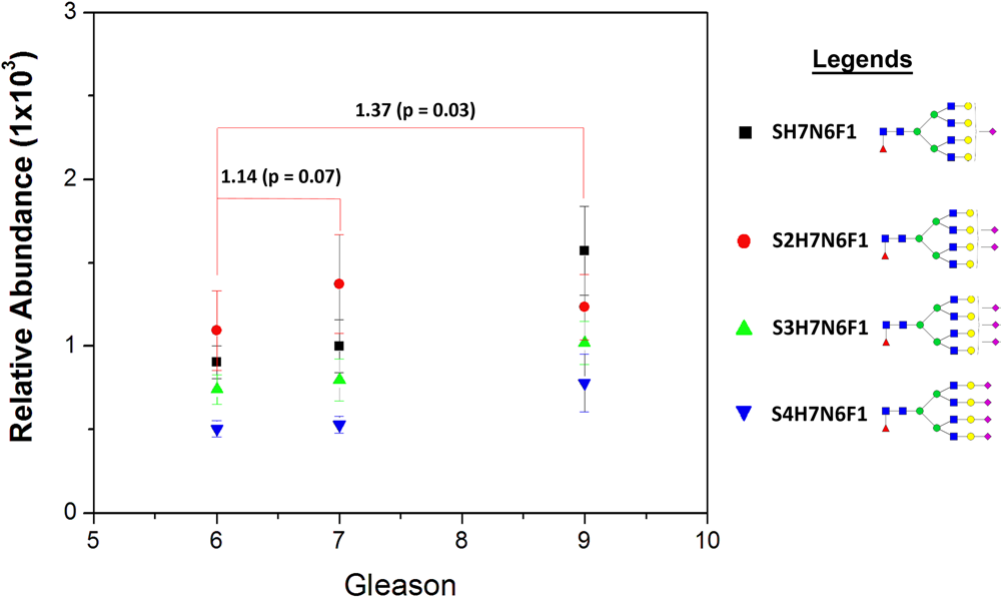
Upregulated fucosylated sialic acid N-glycans in prostate cancer urines. Highly sialylated N-glycans were upregulated in metastatic cancer patients.

Highly branched fucosylated sialic acids are upregulated in metastatic prostate cancer urines^49^. As shown in Fig. 7, four complex fucosylated N-glycans: S1H7N6F1, S2H7N6F1, S3H7N6F1, and S4H7N6F1, were more abundant in Gleason 9 (1.37 fold, p = 0.03) patients compared to Gleason 7 and Gleason 6 patients, confirming the previous findings and demonstrating the potential of the developed method to detect these changes. The results were based on data from 37 of Gleason 6, 15 of Gleason 7, and 6 of Gleason 9. These N-glycans are fucosylated with at least one sialic acid. It has been reported that core fucosylation and α2–3 sialylation is significantly increased in prostate cancer in patient serum^49^. Serum N-glycan analysis showed that biantennary core-fucosylated sialic acid (S1H5N4F1) was highly overexpressed in cancer patients, while the highly branched N-glycans such as S4H7N6F1 have negligible change in cancer vs. benign. Another study demonstrated that high-mannose and fucosylated biantennary complex N-glycans are observed in the serum of prostate cancer patients^50^. Moreover, it has been reported that the urinary free prostate specific antigen (fPSA) is upregulated in metastatic prostate cancer^51, 52^. The up-regulation of the fPSA increases the complex sialylated N-glycans, because the sialic acid α2–3 galactose was found to increase in the prostate cancer compared to the benign^53^. The increased on sialylation of prostate cancers may be attributed to the up-regulation of glycosylation enzymes such as ST3Gal1 and ST6GalNAc1^54, 55^, however, further studies incorporating global proteomics are necessary to confirm these hypothesis.

Using the AutoTip technique, we are able to analyze 58 urine samples derived from prostate cancer patients with varied Gleason scores. In contrast to using a snap-cap spin-column or batch format, which may prove challenging when working with large sample sets, as well as being time-consuming, the reproducible, automated procedure described in this study enabled us to analyze a large number of complex specimens.

## Conclusion

The results of our study have demonstrated that analysis of a large number of samples is feasible using AutoTip format in conjunction with an automated liquid handler. It is prerequisite that the platform must be robust and reliable for generation of accurate results. Distinct from other approaches, we utilized a chemoenzymatic method to immobilize proteins for HTP analysis of N-glycans.

AutoTip automation has several unique advantages over manual processing of samples using the same chemoenzymatic strategy. First, the routine method, that uses snap-cap spin column (SCSC) for manual preparation, can be utilized for processing a limited number of samples simultaneously^25^. In addition, it takes up to 3 days for sample preparation using SCSC, even when reducing the number of washes that are performed between steps (yet still obtaining effective sample clean-up). To summarize, AutoTips can process up to 96 samples at the same time; taking approximately about 1 day when incorporating six washes between steps. The AutoTips methodology is highly reproducible, achieving a CV less than 20%. Automation is one of major advantages using AutoTip, over manual preparatory strategies, for the analysis of glycan and protein profiles from a large number of biological samples. To demonstrate the feasibility of this approach for analyzing a large sample set, we profiled N-glycans from 58 urine samples, revealing sialylated N-glycan glycoform dominated the urine glycomic profile of prostate cancer patients. The profile of N-glycans using AutoTip is similar to that of permethylation (Supplementary Figure S5). Additionally, this platform can be further adapted and utilized for analysis of global proteomics, as well as intact glycoproteomics. There are a few things to be cautious when using the AutoTip platform: (a) resin leakage during operation could potentially lead to sample loss, thus it is recommended to fix the filter in the AutoTip after packing; and (b) the capacity of protein on resin is limited by the volume of AutoTip. A large tip is suggested to use for immobilization of a large amount of proteins (such as > 2 mg). Optionally, proteins can be also aliquot in several tips to increase final N-linked glycan yields. Overall, we have shown that AutoTip is a robust platform for the analysis of N-linked glycans, and has applications for HTP analysis of biological samples.

## Methods

### Materials and Chemicals

Chemicals were purchased from Sigma-Aldrich (St. Louis, MO) unless specified otherwise. Aminolink resin and SCSC were purchased from Life Technologies (Grand Island, NY). Alltech extract clean Carbograph were from Grace (Columbia, MD). Hypercarb AutoTip (TT2CAR; 10–200 μL) was packed by Glygen Corporation (Columbia, MD). C18 analytical LC column (NanoViper, 75 μm, 150 mm, 2 μm particle size) were from Fisher Scientific (Waltham, MA). Eppendorf 96-well plate, matrix D.A.R.T.s tips (20–300 μL), automation reservoirs were purchased from Thermo Fisher Scientific (Hudson, NH). Denaturing buffer (10×), GlycoBuffer (10×), and Peptide-N-glycosidase F (PNGase F) were from New England Biolabs (Ipswich, MA). Trypsin gold (mass spectrometry (MS) grade) were ordered from Promega Corporation (Madison, WI). The polyethylene sheet has typical median pore size 15–45 μm (thickness = 1.57 mm or 0.062 in; Interstate Specialty Products; Sutton, MA).

### Protein Immobilization using Snap-Cap Spin Column (SCSC)

Aminolink resin was loaded onto the SCSC, centrifugation at 2000 × g for 30 sec. Resin was conditioned by pH 10 buffer (500 μL; 100 mM sodium citrate and 50 mM sodium carbonate), repeat twice. Fetuin (200 μg) was mixed with Aminolink resin in SCSC. The supernatant (20 μL) was taken out after 10, 30, 100, 300, and 1440 min. The protein concentration in the supernatant was determined by BCA assay, and the ratio of the remained proteins in solution vs. that prior to immobilization was calculated.

### N-glycan release from Aminolink resin using SCSC

The dynamic of N-glycan release from the resin was conducted using bovine fetuin. Following protein immobilization, sample was reduced in 1x PBS in the presence of 50 mM NaCNBH_3_ (500 μL) for 3 h. After aldehyde sites on Aminolink resin were blocked by 1 M Tris-HCl in the presence of 50 mM NaCNBH_3_, N-glycans were cleaved from the resin by 1 μL PNGase F in freshly prepared ammonium bicarbonate (25 mM). N-glycans were collected in supernatant after 25, 50, 100, 720, and 1440 min at 37 °C.

### Urine sample preparation

Urine samples from prostate cancer patients were provide by the Johns Hopkins University Department of Urology and all methods were performed in accordance with guidelines and regulations with the approval of the Institutional Review Board of Johns Hopkins University. The urine samples were stored in −80 °C before use. The urine typically consists of water (91–96%), inorganic salts, and organic compounds. Some compounds such as urea contain amide groups or amine-containing metabolites that will interfere with the Aminolink resin. Thus those inorganic salts must be removed from sample for protein conjugation. Similar to our previous study^56^, after being thawed in room temperature, the inorganic salts in urines (3–4 mL) were removed using centriprep centrifugal filter (NMWL of 10 kDa; EMD Millipore; Billerica, MA). Briefly, urines were loaded to the centriprep filter and centrifuged at 3000 × g for 20 min; the lysis buffer was prepared consisting of 20 mM PBS (pH 7.4), 150 mM NaCl, 1 mM Na_2_EDTA, 1 mM EGTA, 1% NP40, 2.5 mM sodium pyrophosphate, 1 mM β-glycerophosphate, 1 mM Na_3_VO_4_, and 1 μg/ml leupeptin. A total volume of 3 mL lysis buffer was added to the filter for centrifugation at 3000 × g for another 15~20 min. The ideal final volume should be less than 500 μL since the volume for a 96-well plate is less than 800 μL. If protein concentration is low due to the large volume remained, additional centrifugation (5~10 min) can be further conducted.

### AutoTip fabrication

The detailed protocol using GIG has been described previously^25, 26^. We used Thermo Scientific matrix D.A.R.T.s tips (maximum volume = 300 μL). As shown in Fig. 3a, AutoTip packing starts from insertion of a 2 mm polyethylene sheet, which is cut by 2 mm diameter of Harris Uni-Core biopsy punch (Ted Pella, Inc.; Redding, CA); the Aminolink resin or Hypercarb particles (Glygen; Columbia, MD) (~200 μL) was loaded to the AutoTip, followed by dispensing storage buffer; the second polyethylene sheet (5 mm in diameter) was inserted to seal the AutoTip (Harris Uni-Core biopsy punch 5.0) (Note: the D.A.R.T.s tip can contain up to 300 μL solution; a larger tip may be required if over 300 μL volume is used). The packed AutoTip is ready for use in the Thermo Scientific Versette Liquid Handler.

### AutoTip automation

N-glycans can be studied using the AutoTip. Figure 3b shows the schematic workflow for analysis of N-glycans. The resin is mesoporous particles in which the outer and inner surface is functionalized with aldehyde groups. The liquid handler aspirated sample to the resin and forced proteins passing through the pores; N-termini or lysines fully react with aldehydes on resin surface so that the efficiency for protein conjugation was improved if other factors remain same such as reaction time and temperature. The proteins immobilized inside the pores can fully react with chemicals by aspirating solution through the pores; therefore, modification of sialic acid residues or aspartic acid (D)/glutamic acid (E) is enhanced by thoroughly contacting to the immobilized proteins. Modification of sialic acids was performed by adding 400 μL of 1 M p-Toluidine in 1 M HCl, 40 μL EDC (1-Ethyl-3-(3-dimethylaminopropyl) carbodiimide), and 25 μL HCl (36–38%). The reaction was proceed at room temperature for 3 h. N-glycans were released by PNGase F digestion, or PNGase A if glycopeptides were used. The released N-glycans were purified by Hypercarb AutoTip using same liquid handler. The Hypercarb AutoTip was packed with porous graphitized particles. These procedures enable to identify N-glycans.

### AutoTip N-glycan release

The N-glycans were released by 1 μL PNGase F, 30 μL GlycoBuffer (10×), and 270 μL DI water, incubation at room temperature for 3 h. The supernatant was collected in 96-well deep-well microplate (Eppendorf; VWR; Radnor, PA). The AutoTip was washed with 150 μL DI water to collect supernatant, repeat once. All supernatants were combined and formic acid (100%) was added to adjust pH less than 3. The Hypercarb AutoTip was placed in the liquid handler and pre-conditioned by 80% acetonitrile (ACN) (300 μL; 5 cycles) and 10% trifluoroacetic acid (TFA) (300 μL; 5 cycles); samples (~600 μL) were loaded to the Hypercarb AutoTip (40 cycles); the AutoTips were further washed by 0.1% FA for 20 cycles; N-glycans were finally eluted using 300 μL elution solution (80% ACN in 0.1% FA). Samples were dried in a Speed-Vac and resuspended in 100 μL 0.2% FA, from which 4 μL samples were tested by MALDI-MS for structural analysis of N-glycans. MALDI matrix consists of 100 mg/mL DHB (2,5-Dihydroxybenzoic acid), 1 μL DMA (N,N-Dimethylaniline) per 50 μL matrix solution at 50% ACN in presence of 0.1 mM NaCl^25^. N-glycans were searched against glycomic databases^57^ including CFG (Consortium Functional Glycomics), CarbBank^58^, GlycomeDB^59^, and Glycosciences^60^ using GlycoWorkbench^61^. A list of potential N-glycans were also generated according to the accurate precursor mass.

### MALDI-MS

MALDI-MS was conducted on a Shimadzu MAXIMA Resonance with a mass range from 850 to 6000 Da. The MALDI matrix consisted of 100 mg/mL DHB in the presence of 0.1 mM NaCl and 25 μM DP7 which is used as an internal standard for semi-quantitation^62^. The DMA was added at a ratio of 0.02 μL per μL of matrix solution^33^. For un-derivatized glycan, each sialic acid carries one sodium abduct ions, e.g., S1H5N4 = [M-H + 2Na]^+^ (1976.2 Da). Glycan composition was determined by precursor library matching and verified by MS/MS if necessary.

### QUANTITY labeling of N-glycans

The purified N-glycans were labeled with isobaric tags by reducing-ends for the relative quantification. The released N-glycans by PNGase F has a non-reducing end that was utilized for reductive amination with QUANTITY^28^. Briefly, the purified N-glycans were dried in Speed-Vac before mixing with 20 μL 1 M NaCNBH_3_ in DMSO-AA (acetic acid) (7:3, vol) and 20 μL 100 mM QUANTITY tag (4 plex includes 176, 177, 178, and 179). Samples were briefly vortexed and centrifuged in 2 mL Eppendorf tube. The mixture was heated at 65 °C for 4 h. The labeled N-glycans were pooled (~80 μL) and diluted to the total volume of 8 mL (or at least 25-fold dilution). The pH of diluted N-glycan sample was lowered by adding 8 μL of formic acid and cleaned by Carbograph. The elute was dried in Speed-Vac and re-suspended in 0.2% FA for quantitative analysis by LC-MS/MS.

### N-glycan LC-ESI-MS/MS

The labeled N-glycans were tested using Thermo Orbitrap Velos. The LC gradient was set as following: 0–5 min, 96% HPLC water (0.1% FA) (A) and 4% HPLC ACN (0.1% FA) (B); 5–100 min, 4–50% B; 100–105, 50–95% B; 115–116, 95–4% B, 116–120, 4% B. Flow rate was fixed at 0.3 µL/min. The MS setting for Velos was set as following: (a) MS1: resolution was 60,000, CID (collision-induced dissociation) energy 35, mass range 400–1800 (m/z); (b) MS/MS: resolution was 7,500, rejected charge 1, high-energy collisional dissociation activation energy 35, isolation width (m/z) 4, and activation time 0.2 ms.

## Electronic supplementary material

AutoTip Supplementary Materials

Table S1

Table S2_run1

Table S2_run2

Table S2_run3

Table S3

Table S4

## References

[1] Ohtsubo K, Marth JD (2006). Glycosylation in cellular mechanisms of health and disease. Cell.

[2] Arnold JN, Wormald MR, Sim RB, Rudd PM, Dwek RA (2007). The impact of glycosylation on the biological function and structure of human immunoglobulins. Annu. Rev. Immunol..

[3] Hakomori S (2002). Glycosylation defining cancer malignancy: new wine in an old bottle. Proc. Natl. Acad. Sci. USA.

[4] Pinho SS, Reis CA (2015). Glycosylation in cancer: mechanisms and clinical implications. Nat. Rev. Cancer.

[5] Tian Y (2015). Identification of sialylated glycoproteins from metabolically oligosaccharide engineered pancreatic cells. Clin. Proteomics.

[6] Wang X (2014). Overexpression of α (1, 6) fucosyltransferase associated with aggressive prostate cancer. Glycobiology.

[7] Stahl-Zeng J (2007). High sensitivity detection of plasma proteins by multiple reaction monitoring of N-glycosites. Mol. Cell Proteomics.

[8] Dalpathado DS, Desaire H (2008). Glycopeptide analysis by mass spectrometry. Analyst.

[9] Lau KS (2007). Complex N-glycan number and degree of branching cooperate to regulate cell proliferation and differentiation. Cell.

[10] Goldberg D, Sutton‐Smith M, Paulson J, Dell A (2005). Automatic annotation of matrix‐assisted laser desorption/ionization N‐glycan spectra. Proteomics.

[11] Leymarie N, Zaia J (2012). Effective use of mass spectrometry for glycan and glycopeptide structural analysis. Anal. Chem..

[12] Yang W (2014). Glycoproteomic analysis identifies human glycoproteins secreted from HIV latently infected T cells and reveals their presence in HIV+ plasma. Clin. Proteomics.

[13] Liu Y (2014). Glycoproteomic analysis of prostate cancer tissues by SWATH mass spectrometry discovers N-acylethanolamine acid amidase and protein tyrosine kinase 7 as signatures for tumor aggressiveness. Mol. Cell Proteomics.

[14] Sun S (2015). Comprehensive analysis of protein glycosylation by solid-phase extraction of N-linked glycans and glycosite-containing peptides. Nat. Biotechnol..

[15] Kaji H (2003). Lectin affinity capture, isotope-coded tagging and mass spectrometry to identify N-linked glycoproteins. Nat. Biotechnol..

[16] Drake PM (2011). A lectin affinity workflow targeting glycosite-specific, cancer-related carbohydrate structures in trypsin-digested human plasma. Anal. Biochem..

[17] Zhang H, Li X-j, Martin DB, Aebersold R (2003). Identification and quantification of N-linked glycoproteins using hydrazide chemistry, stable isotope labeling and mass spectrometry. Nat. Biotechnol..

[18] Tian Y, Zhou Y, Elliott S, Aebersold R, Zhang H (2007). Solid-phase extraction of N-linked glycopeptides. Nat. Protoc..

[19] Shubhakar A (2015). High-throughput analysis and automation for glycomics studies. Chromatographia.

[20] Royle L (2008). HPLC-based analysis of serum N-glycans on a 96-well plate platform with dedicated database software. Anal. Biochem..

[21] Cook KS, Bullock K, Sullivan T (2012). Development and qualification of an antibody rapid deglycosylation method. Biologicals.

[22] Burnina I, Hoyt E, Lynaugh H, Li H, Gong B (2013). A cost-effective plate-based sample preparation for antibody N-glycan analysis. J. Chromatogr. A.

[23] Lauber MA (2015). Rapid preparation of released N-glycans for HILIC analysis using a labeling reagent that facilitates sensitive fluorescence and ESI-MS detection. Anal. Chem..

[24] Aich U, Lakbub J, Liu A (2016). State of the art technologies for rapid and high‐throughput sample preparation and analysis of n‐glycans from antibodies. Electrophoresis.

[25] Yang S, Li Y, Shah P, Zhang H (2013). Glycomic analysis using glycoprotein immobilization for glycan extraction. Anal. Chem..

[26] Yang S, Zhang H (2014). Glycomic analysis of glycans released from glycoproteins using chemical immobilization and mass spectrometry. Curr. Protoc. Chem. Biol..

[27] Yang S, Hu Y, Sokoll L, Zhang H (2017). Simultaneous quantification of N-and O-glycans using a solid-phase method. Nat. Protoc..

[28] Yang S (2015). Quantity: an isobaric tag for quantitative glycomics. Sci. Rep..

[29] Shah P (2015). Tissue proteomics using chemical immobilization and mass spectrometry. Anal. Biochem..

[30] Yang S (2015). Integrated glycoprotein immobilization method for glycopeptide and glycan analysis of cardiac hypertrophy. Anal. Chem..

[31] Yang S, Toghi Eshghi S, Chiu H, DeVoe DL, Zhang H (2013). Glycomic analysis by glycoprotein immobilization for glycan extraction and liquid chromatography on microfluidic chip. Anal. Chem..

[32] Chen J, Shah P, Zhang H (2013). Solid phase extraction of N-linked glycopeptides using hydrazide tip. Anal. Chem..

[33] Yang S, Zhang H (2012). Glycan analysis by reversible reaction to hydrazide beads and mass spectrometry. Anal. Chem..

[34] Abdel-Magid AF, Carson KG, Harris BD, Maryanoff CA, Shah RD (1996). Reductive amination of aldehydes and ketones with sodium triacetoxyborohydride. studies on direct and indirect reductive amination procedures 1. J. Org. Chem..

[35] Ruhaak L (2010). Glycan labeling strategies and their use in identification and quantification. Anal. Bioanal. Chem..

[36] Guttman A, Chen F-TA, Evangelista RA, Cooke N (1996). High-resolution capillary gel electrophoresis of reducing oligosaccharides labeled with 1-aminopyrene-3, 6, 8-trisulfonate. Anal. Biochem..

[37] Chen F-TA, Dobashi TS, Evangelista RA (1998). Quantitative analysis of sugar constituents of glycoproteins by capillary electrophoresis. Glycobiology.

[38] Yang S (2017). Modification of sialic acids on solid-phase: accurate characterization of protein sialylation. Anal. Chem..

[39] Pabst M, Altmann F (2008). Influence of electrosorption, solvent, temperature, and ion polarity on the performance of LC-ESI-MS using graphitic carbon for acidic oligosaccharides. Anal. Chem..

[40] Sekiya S, Wada Y, Tanaka K (2005). Derivatization for stabilizing sialic acids in MALDI-MS. Anal. Chem..

[41] Shah P (2013). Mass spectrometric analysis of sialylated glycans with use of solid-phase labeling of sialic acids. Anal. Chem..

[42] Wheeler SF, Domann P, Harvey DJ (2009). Derivatization of sialic acids for stabilization in matrix‐assisted laser desorption/ionization mass spectrometry and concomitant differentiation of α (2 → 3)‐and α (2 → 6)‐isomers. Rapid Commun. Mass Spectrom..

[43] Yang, S., Jankowska, E., Kosikova, M., Xie, H. & Cipollo, J. F. Sialic acid linkage analysis of influenza virus glycoproteins using solid-phase chemical modification. *Anal. Chem*. In press (2017). 10.1021/acs.analchem.7b02514 28792205

[44] Huang Y, Nie Y, Boyes B, Orlando R (2016). Resolving Isomeric Glycopeptide Glycoforms with Hydrophilic Interaction Chromatography (HILIC). J. Biomol. Tech..

[45] Ahn J, Bones J, Yu YQ, Rudd PM, Gilar M (2010). Separation of 2-aminobenzamide labeled glycans using hydrophilic interaction chromatography columns packed with 1.7 μm sorbent. J. Chromatogr. B.

[46] Guttman A, Chen FTA, Evangelista RA (1996). Separation of 1‐aminopyrene‐3, 6, 8‐trisulfonate‐labeled asparagine‐linked fetuin glycans by capillary gel electrophoresis. Electrophoresis.

[47] Green ED, Adelt G, Baenziger J, Wilson S, Van Halbeek H (1988). The asparagine-linked oligosaccharides on bovine fetuin. Structural analysis of N-glycanase-released oligosaccharides by 500-megahertz 1H NMR spectroscopy. J. Biol. Chem..

[48] Bendiak B, Harris-Brandts M, Michnick SW, Carver JP, Cumming DA (1989). Separation of the complex asparagine-linked oligosaccharides of the glycoprotein fetuin and elucidation of three triantennary structures having sialic acids linked only to galactose residues. Biochemistry.

[49] Saldova R, Fan Y, Fitzpatrick JM, Watson RWG, Rudd PM (2011). Core fucosylation and α2-3 sialylation in serum N-glycome is significantly increased in prostate cancer comparing to benign prostate hyperplasia. Glycobiology.

[50] De Leoz MLA (2008). Glycomic approach for potential biomarkers on prostate cancer: profiling of N-linked glycans in human sera and pRNS cell lines. Dis. Markers.

[51] Catalona WJ (1998). Use of the percentage of free prostate-specific antigen to enhance differentiation of prostate cancer from benign prostatic disease: a prospective multicenter clinical trial. JAMA.

[52] Vermassen T (2015). Urinary prostate protein glycosylation profiling as a diagnostic biomarker for prostate cancer. Prostate.

[53] Ohyama C (2004). Carbohydrate structure and differential binding of prostate specific antigen to Maackia amurensis lectin between prostate cancer and benign prostate hypertrophy. Glycobiology.

[54] Dube DH, Bertozzi CR (2005). Glycans in cancer and inflammation-potential for therapeutics and diagnostics. Nat. Rev. Drug Discov..

[55] Munkley J, Mills IG, Elliott DJ (2016). The role of glycans in the development and progression of prostate cancer. Nat. Rev. Urol..

[56] Jia X (2016). Detection of Aggressive Prostate Cancer‐Associated Glycoproteins in Urine using Glycoproteomics and Mass Spectrometry. Proteomics.

[57] Hizal DB (2014). Glycoproteomic and glycomic databases. Clin. Proteomics.

[58] Doubet S, Albersheim P (1992). Letter to the glyco-forum CarbBank. Glycobiology.

[59] Ranzinger R, Herget S, von der Lieth C-W, Frank M (2011). GlycomeDB-a unified database for carbohydrate structures. Nucleic Acids Res..

[60] Lütteke T (2006). GLYCOSCIENCES.de: an Internet portal to support glycomics and glycobiology research. Glycobiology.

[61] Ceroni A (2008). GlycoWorkbench: a tool for the computer-assisted annotation of mass spectra of glycans†. J. Proteome Res..

[62] Gil G-C, Kim Y-G, Kim B-G (2008). A relative and absolute quantification of neutral N-linked oligosaccharides using modification with carboxymethyl trimethylammonium hydrazide and matrix-assisted laser desorption/ionization time-of-flight mass spectrometry. Anal. Biochem..

