# Supplementary material for: High-throughput analysis of N-glycans using AutoTip via glycoprotein immobilization
Source: Sci Rep. 2017 Aug 31;7:10216. doi: 10.1038/s41598-017-10487-8 (PMC5578957; doi:10.1038/s41598-017-10487-8)
Supplement: Supplementary file 1 — AutoTip Supplementary Materials [file 41598_2017_10487_MOESM1_ESM.doc]

**Supplementary Information for**

**High-throughput analysis of N-glycans using AutoTip *via* glycoprotein immobilization**

Shuang Yang^1*^, David Clark^1^, Yang Liu^1^, Shuwei Li^2^, and Hui Zhang^1^

^1^Department of Pathology, Johns Hopkins Medicine, Baltimore, MD

^2^Institute for Bioscience and Biotechnology Research, University of Maryland College Park, Rockville, MD

*Corresponding to Dr. Shuang Yang at [jake.yang@gmail.com](mailto:jake.yang@gmail.com)

**Keywords**

Chemoenzymatic, Glycoproteomics, Solid-Phase, Automation, Mass Spectrometry

**Abstract**

It is often required to use a method that is efficient, rapid, and highly reproducible for analysis of a large number of sample sets. Automation is ideal approach for high-throughput sample preparation. Multi-plexing sample preparation *via* a 96-well plate format has gained population in recent years; however, those methods lack specificity and require multiple chromatographic purification. To overcome these challenges, a chemoenzymatic method has been developed by utilizing protein conjugation on solid-phase. The individual sample was successfully performed in the snap-cap spin-column (SCSC) platform. However, sample preparation using SCSC is time-consuming and lacks reproducibility. In this work, we integrated the chemoenzymatic technique in a pipette tip (AutoTip) that was operated by an automated liquid handler. We established a multi-step protocol involving protein immobilization, sialic acid modification, and N-glycan release. We first optimized our protocol using bovine fetuin as a standard glycoprotein, and assessed the reproducibility of the AutoTip using isobaric tags for relative quantification of N-glycans. We then applied this methodology to profile N-glycans from 58 prostate cancer patient urine samples, revealing that sialic acids were abundant N-glycans in the urine derived from prostate cancer patients. Our results indicated AutoTip has applications for high-throughput sample preparation for studying N-glycans.

**Methods**

**BCA assay.** Pierce BCA protein assay kit was used for measurement of protein concentration. Briefly, BCA protein assay reagent A (180 µL) was mixed with reagent B (20 µL). The mixture of 200 µL was added to 96-well plate (Thermo). To generate a calibration curve, BSA (Thermo) standard solution was prepared with a series of concentration at 2 mg/ml, 1 mg/ml, 0.75 mg/ml, 0.5 mg/ml, 0.25 mg/ml, 0.125 mg/ml, and 0.0625 mg/ml. Each standard (20 µL) was mixed with 200 µL BCA mixture (A + B). Proteins (20 µL) from samples were also mixed with 200 µL BCA mixture, incubation at 37°C for 30 min. The 96-well plate was loaded to a BCA assay reader (Synergy Microplate Reader; BioTek, Winooski, VT, USA). Protein concentration was determined by calibration curve from BSA standards.

Supplementary Table S5. Comparison of AutoTip versus manual snap-cap spin-column (SCSC) for glycan analysis of glycoproteins.

| Comparison | SCSC (Manual) | AutoTip (Automation) |
| --- | --- | --- |
| Number of sample | 1 up to 24 | 1 up to 384 |
| Fabrication | N/A | 30 sec per AutoTip (packing resin) |
| Sample prep time | 3 days for 1 spin-column; 4 days for 24 spin-columns (only 3 washes per step) | 1 days for up to 96 AutoTips (at least 6 wash per step); no additional time is added when more samples are prepared. |
| Reproducibility | Medium | High (CV < 20%) |
| Automation | No | Yes |

**Supplementary Figure S1. MALDI-MS spectra of bi-antennary sialic acids with and without p-Toluidine derivatization.** (**a**) α2,3-linked sialic acid (native); (**b**) α2,6-linked sialic acid (native); (**c**) mixture of α2,3 and α2,63-linked sialic acid after p-Toluidine modification. Both sialic acids are simultaneously derivatized by p-Toluidine carbodiimide coupling.

**
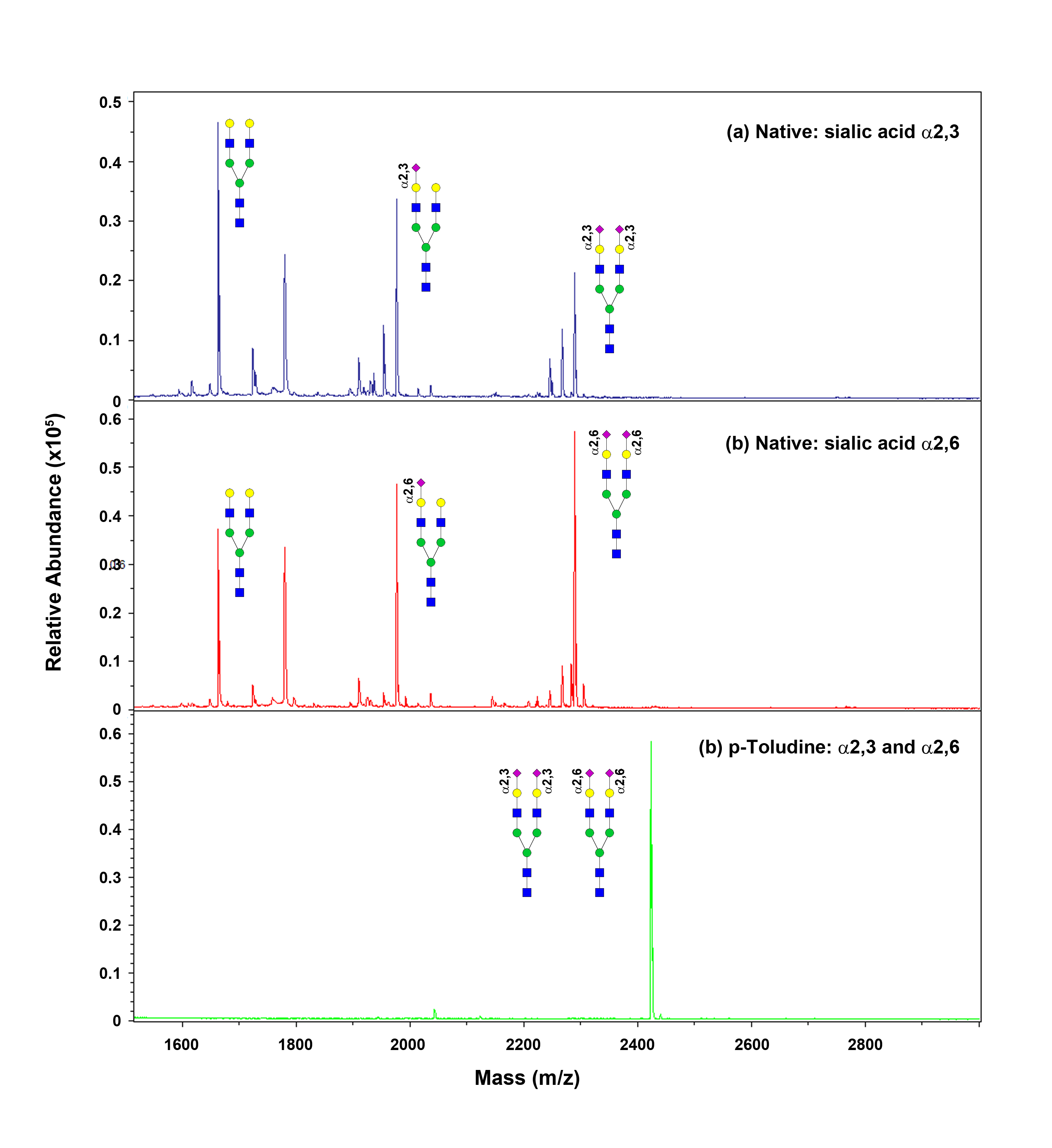
**

**Supplementary Figure S2. Release of N-glycans by PNGase F from the immobilized glycoproteins on AutoTip.** Each AutoTip was conjugated with Bovine Fetuin (1 mg) and released by PNGase F. The flow-through was collected after PNGase F digestion, 2 min, 10 min, 20 min, 60 min, 120 min, and 240 min. (**a**) S1H5N4, (**b**) S2H5N4, (**c**) S2H6N5, and (**d**) S4H6N5.


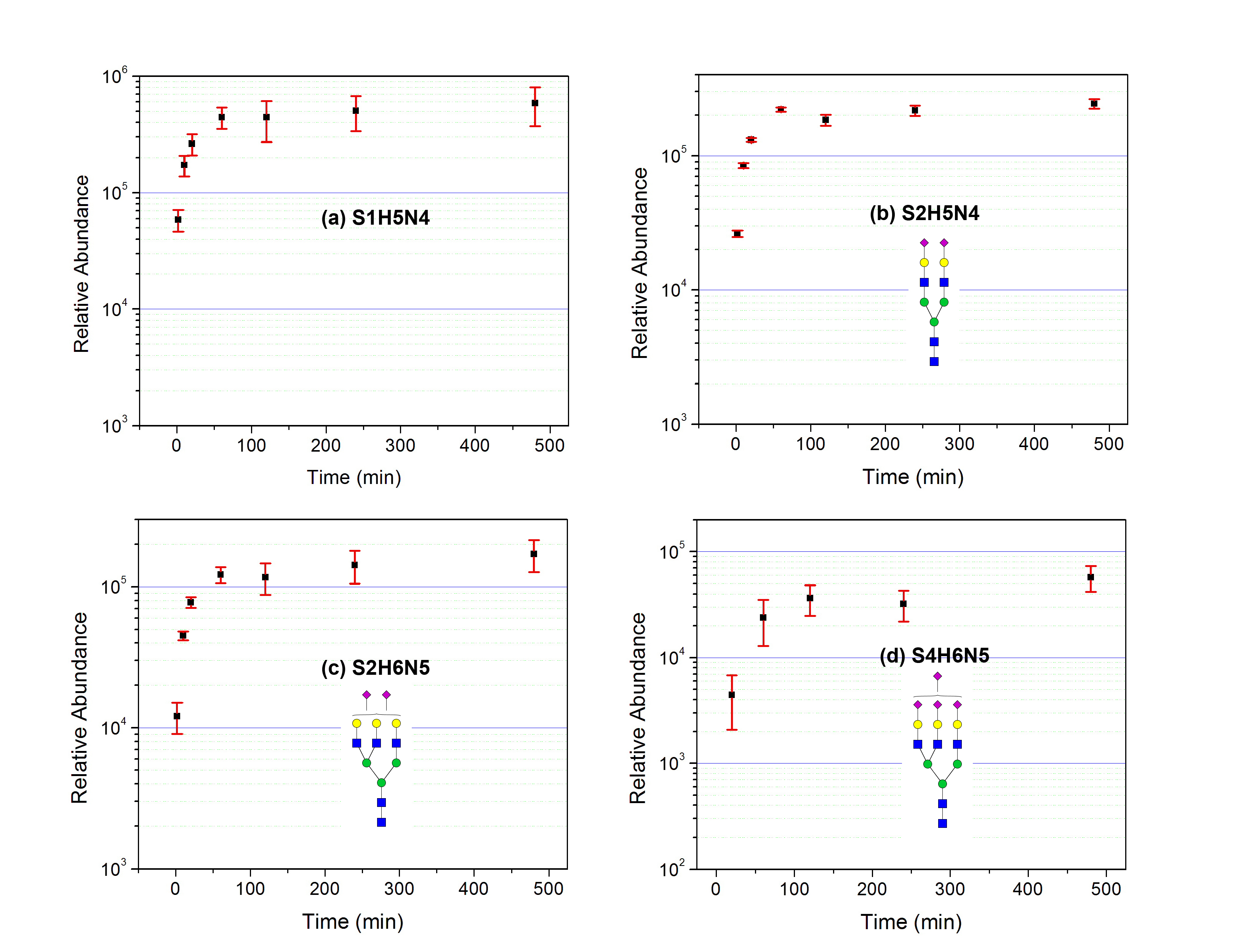


**Supplementary Figure S3. Reproducibility of N-glycans in LC-MS. N-glycans are released from bovine fetuin via AutoTip and labeled with QUANTITY via reductive amination.** Three replicates are conducted on 12 AutoTips. The CV is less than 20%.

**
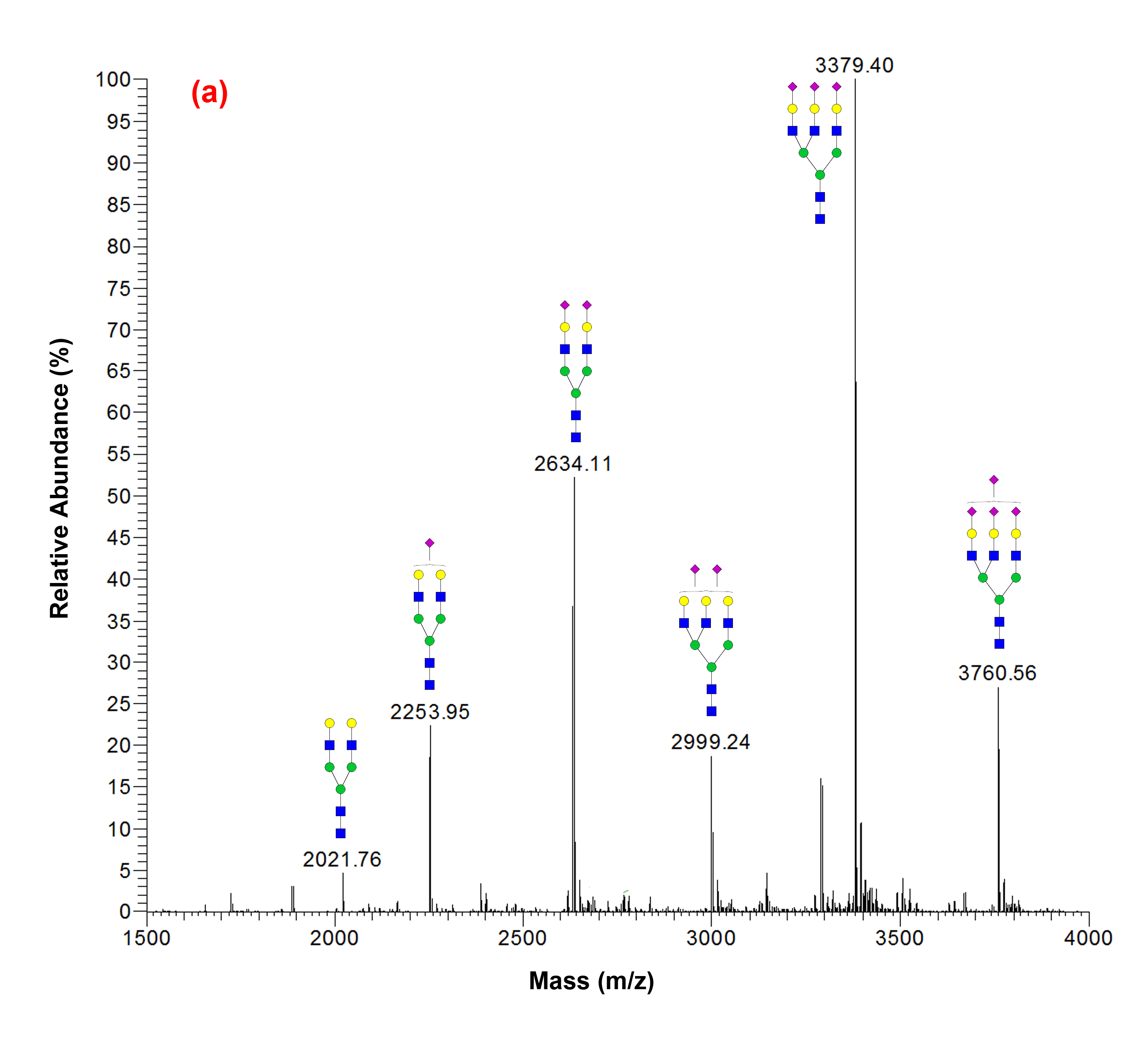
**

**
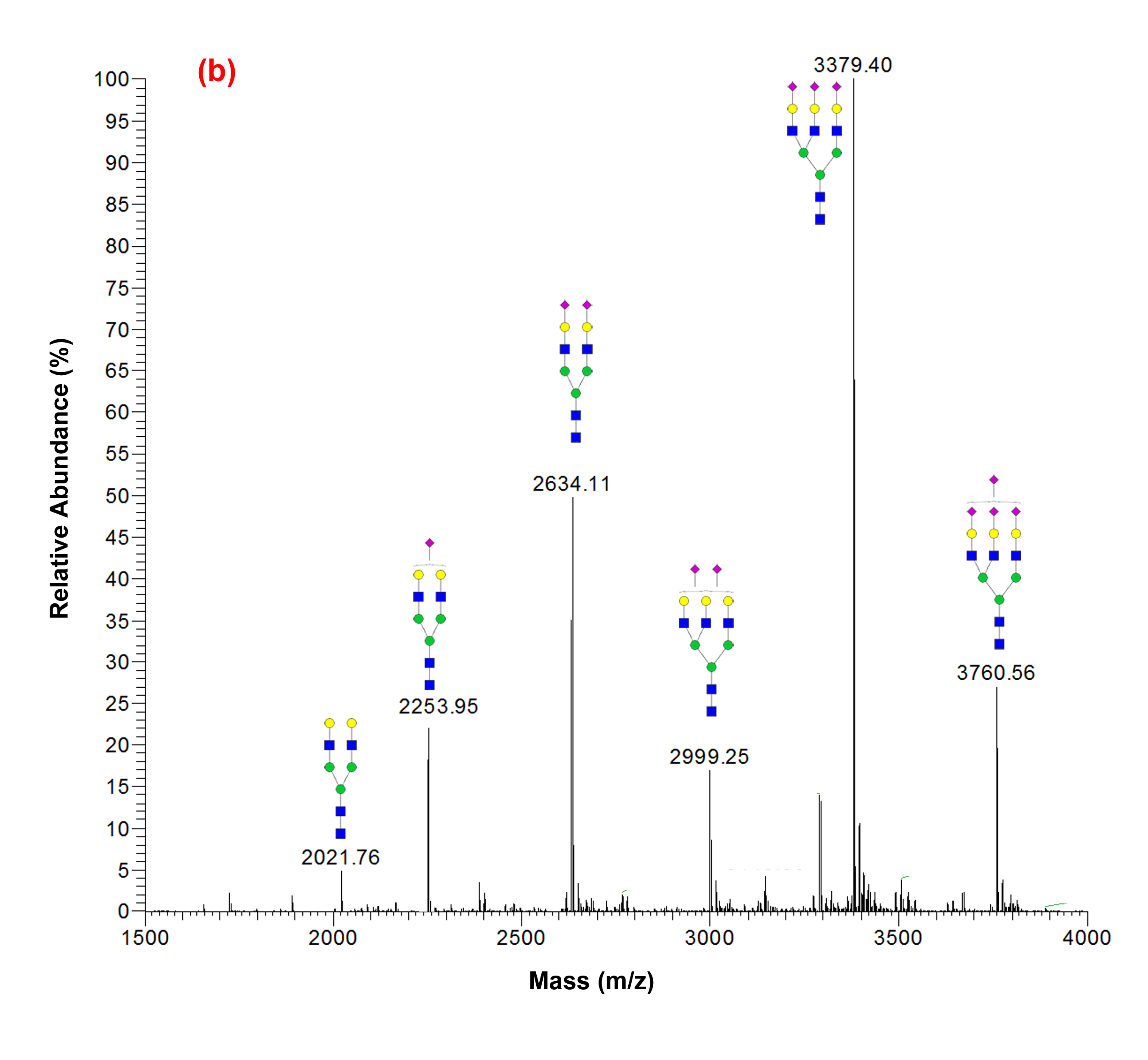

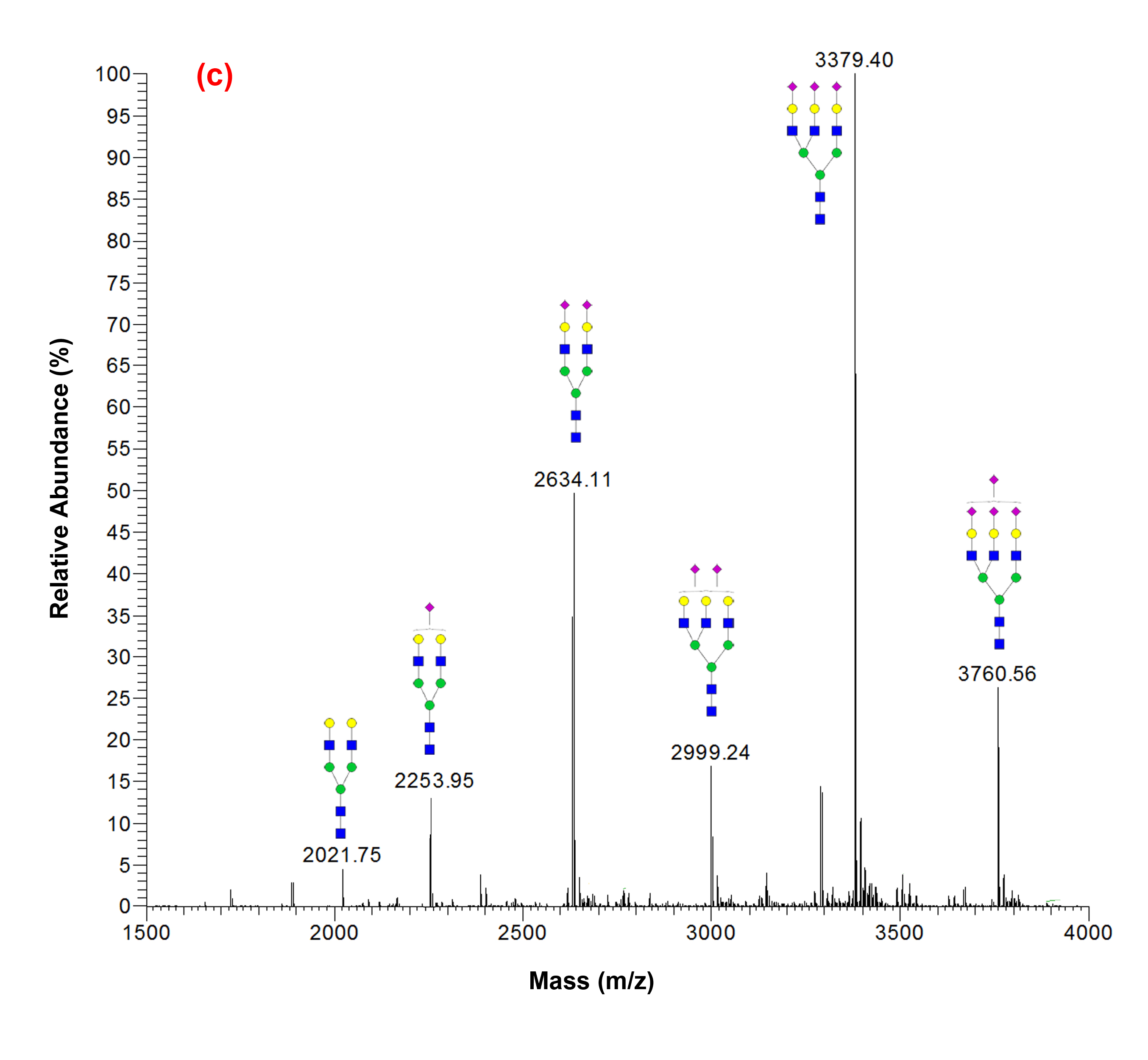
**

**Supplementary Figure S4. MALDI-MS spectra of 58 urine samples with different Gleason scores.** Gleason score 6: sample 1-37 (a,b,c,d,e); Gleason score 7: sample 38-52 (d,e,f); Gleason score 9: sample 53-58 (g). MALDI-MS spectra were produced by Shimadzu Launchpad Biotech.

**
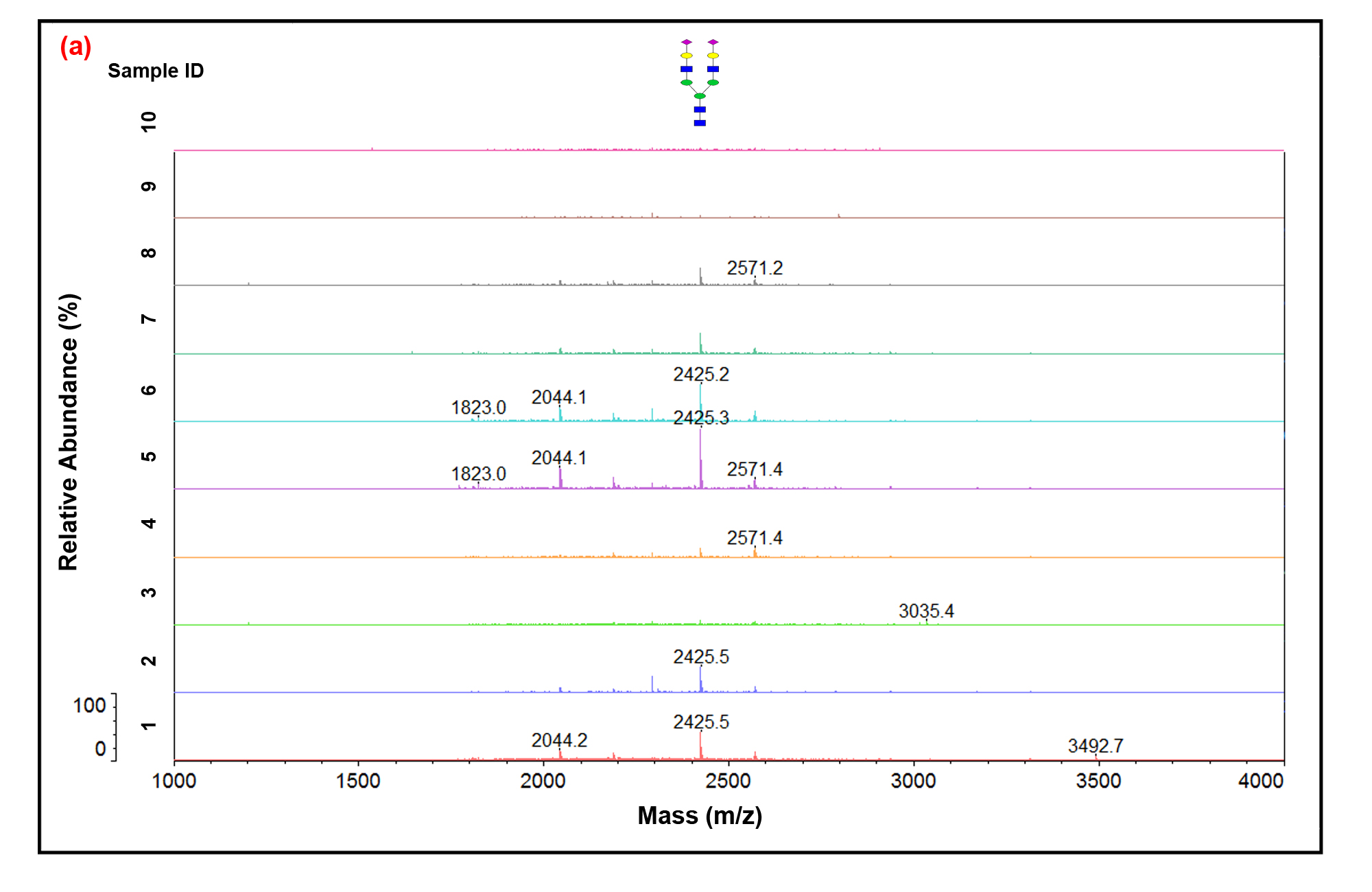

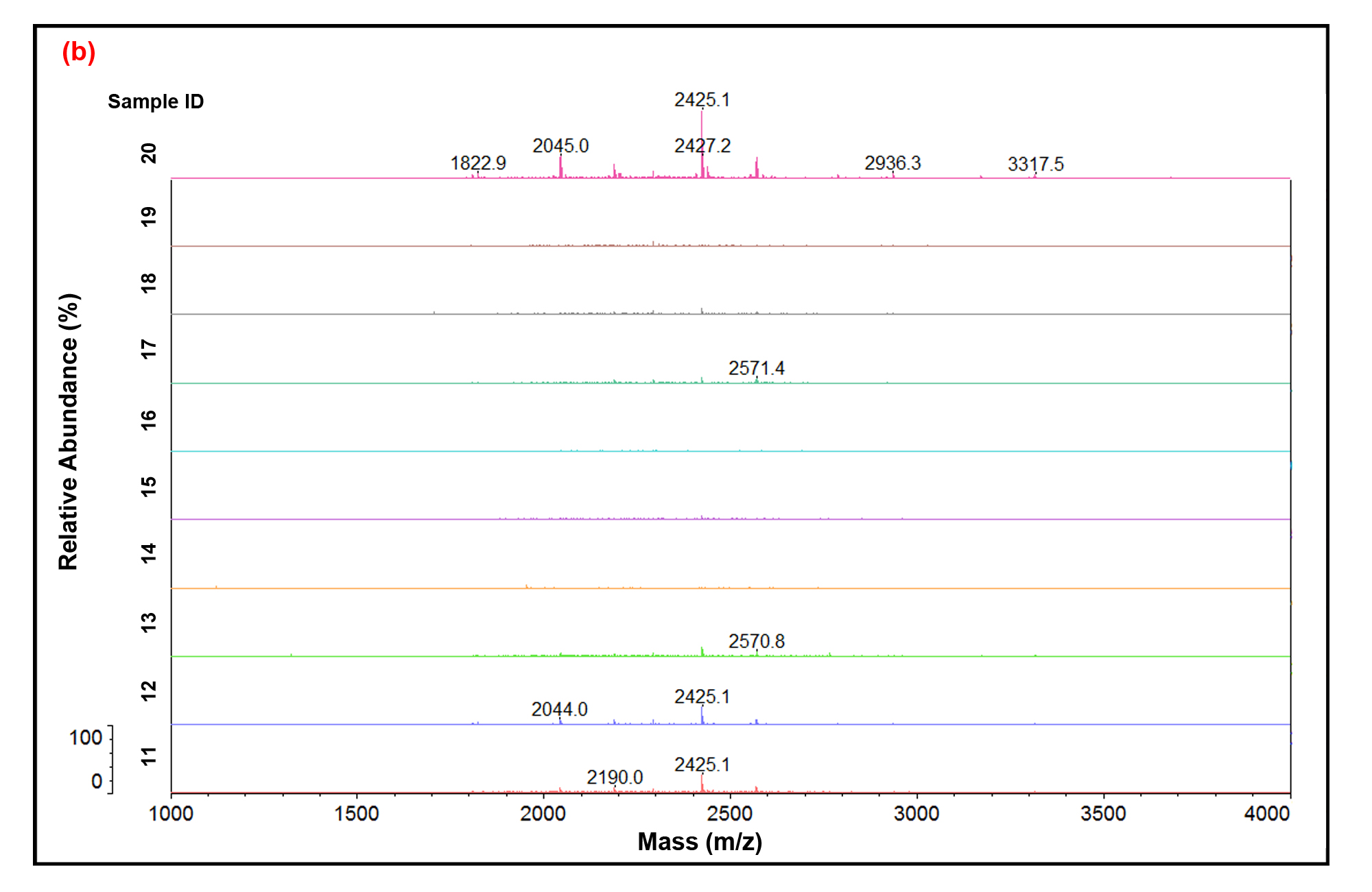
**

**
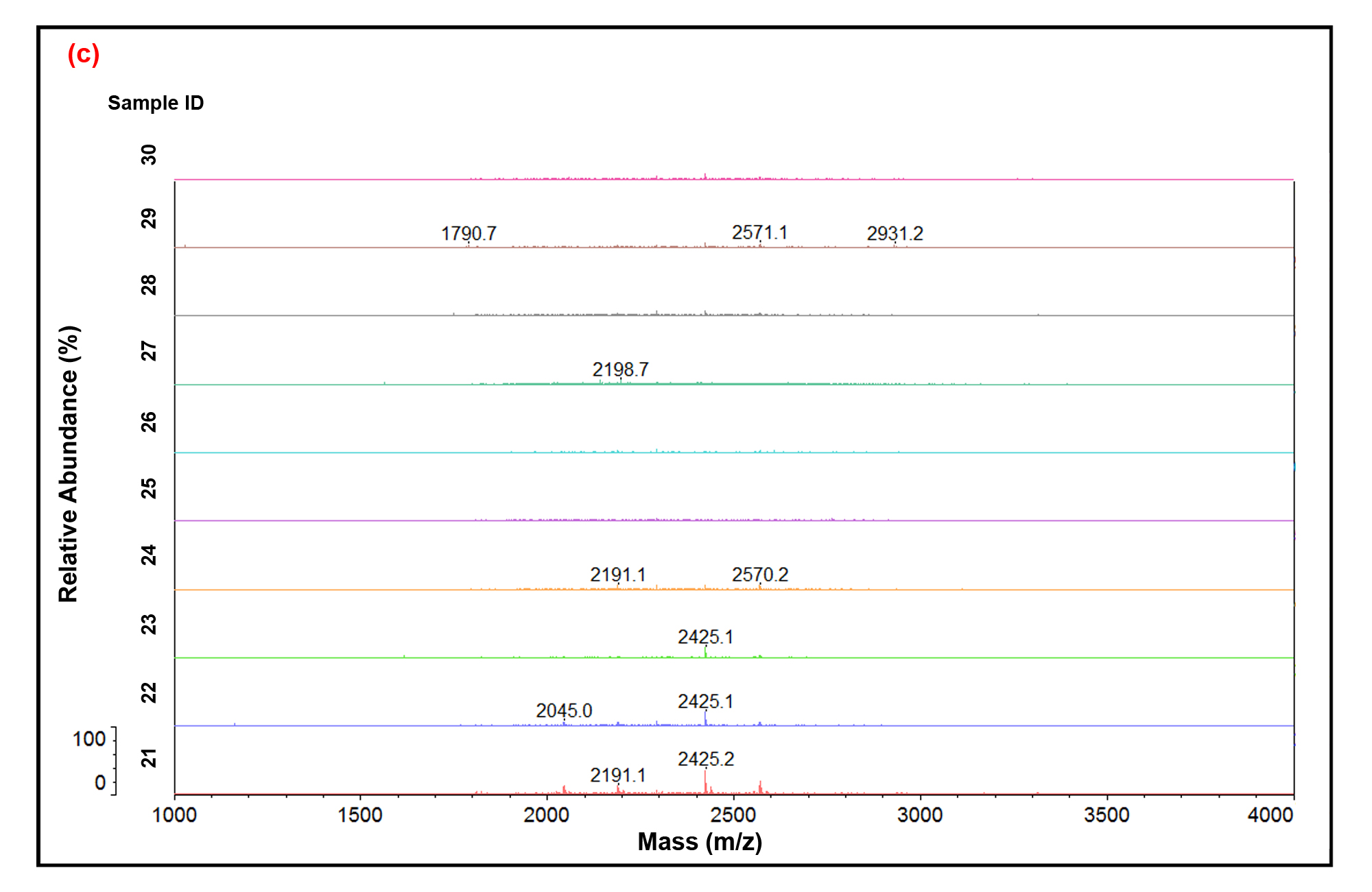

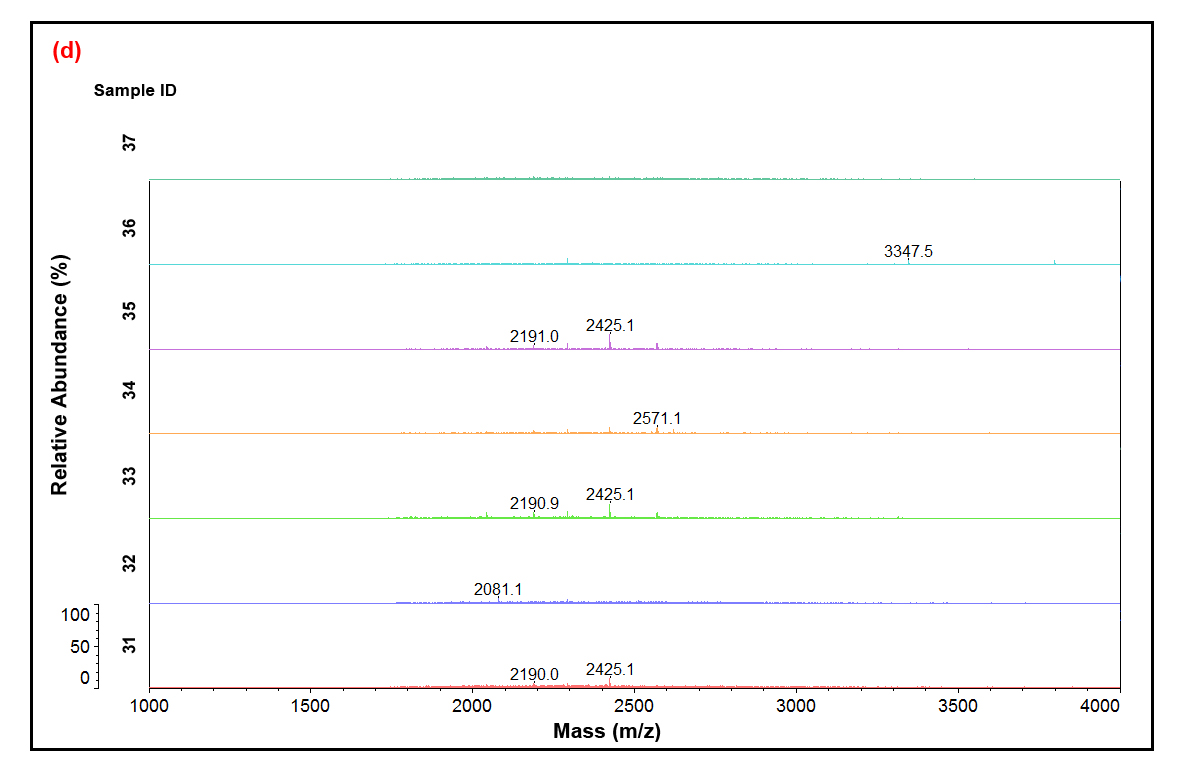
**

**
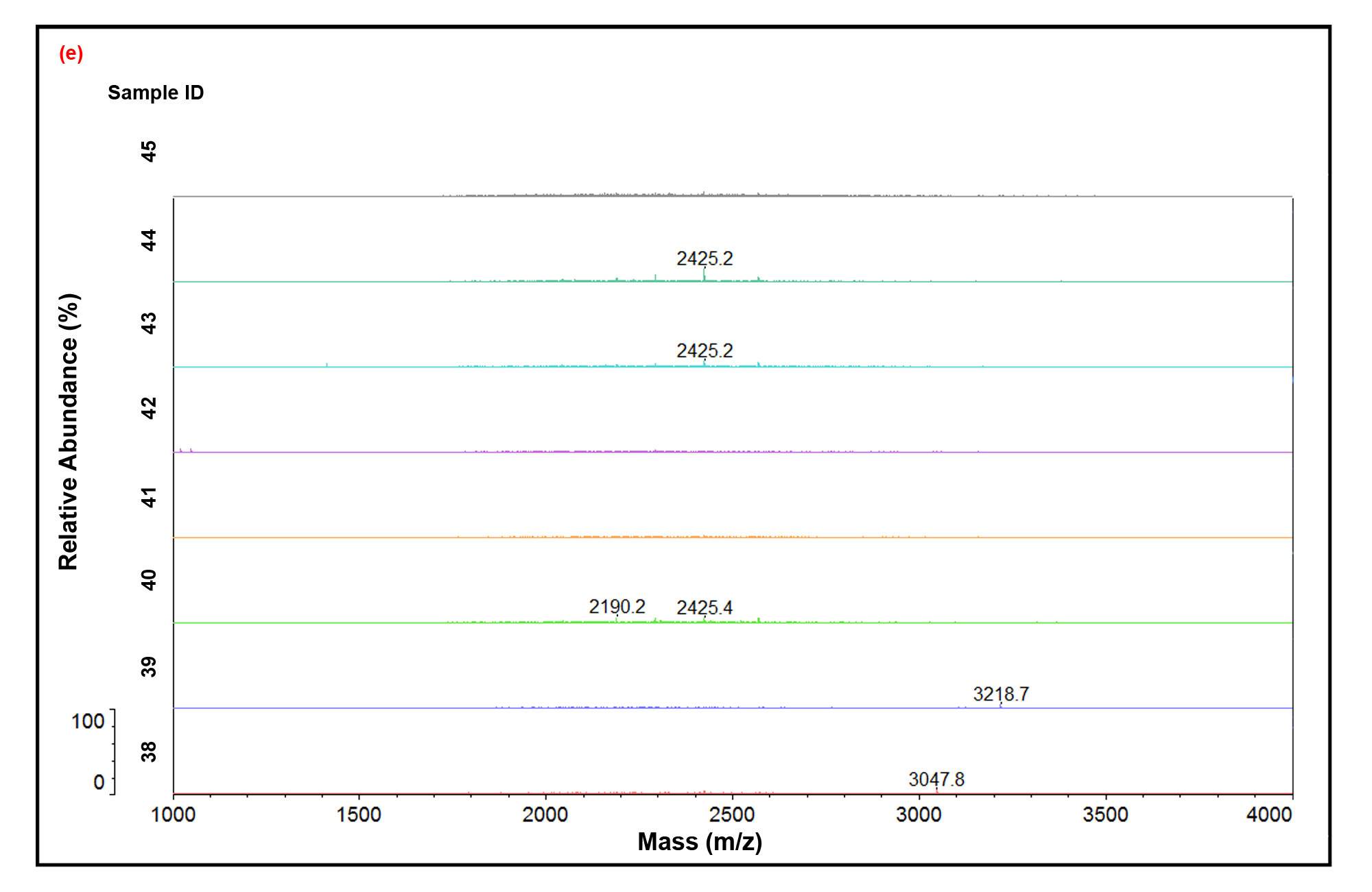

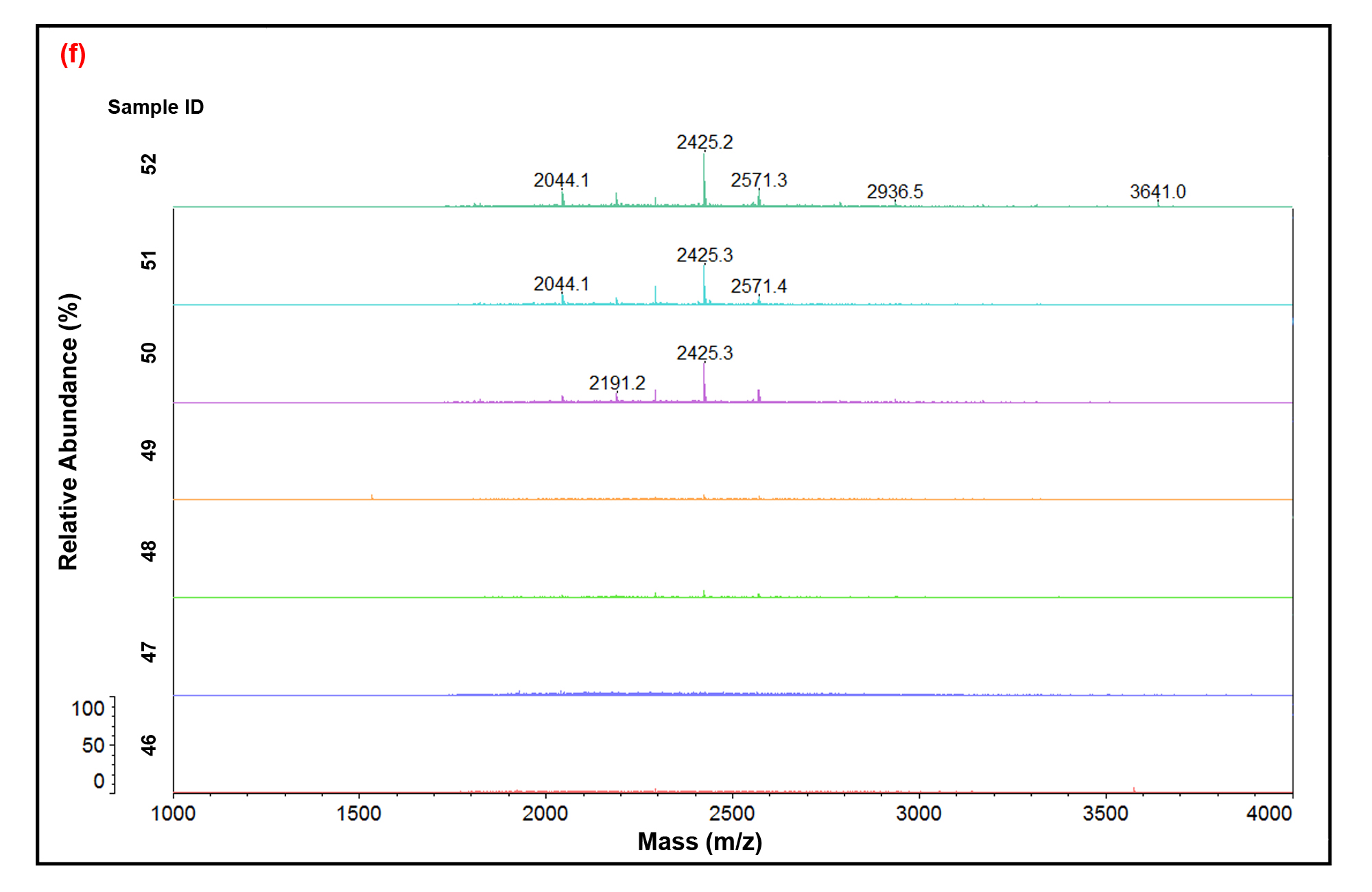
**

**
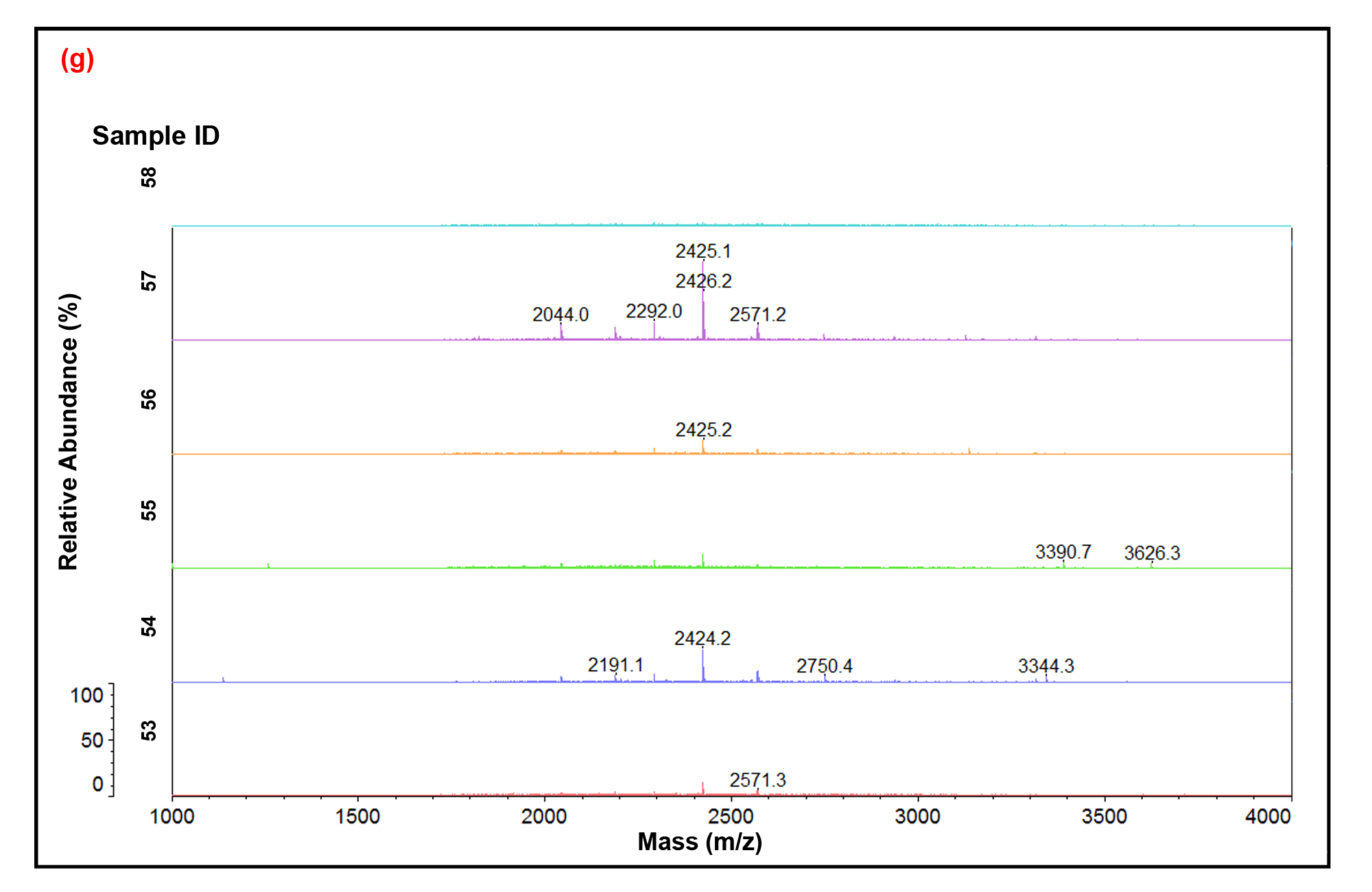
**

**Supplementary Figure S5. MALDI-MS spectra of N-glycans from bovine fetuin.** (**a**) N-glycan released by PNGase F without modification; (**b**) N-glycan modified by p-Toluidine prior to PNGase F digestion; (**c**) N-glycan permethylated after PNGase F release.

**
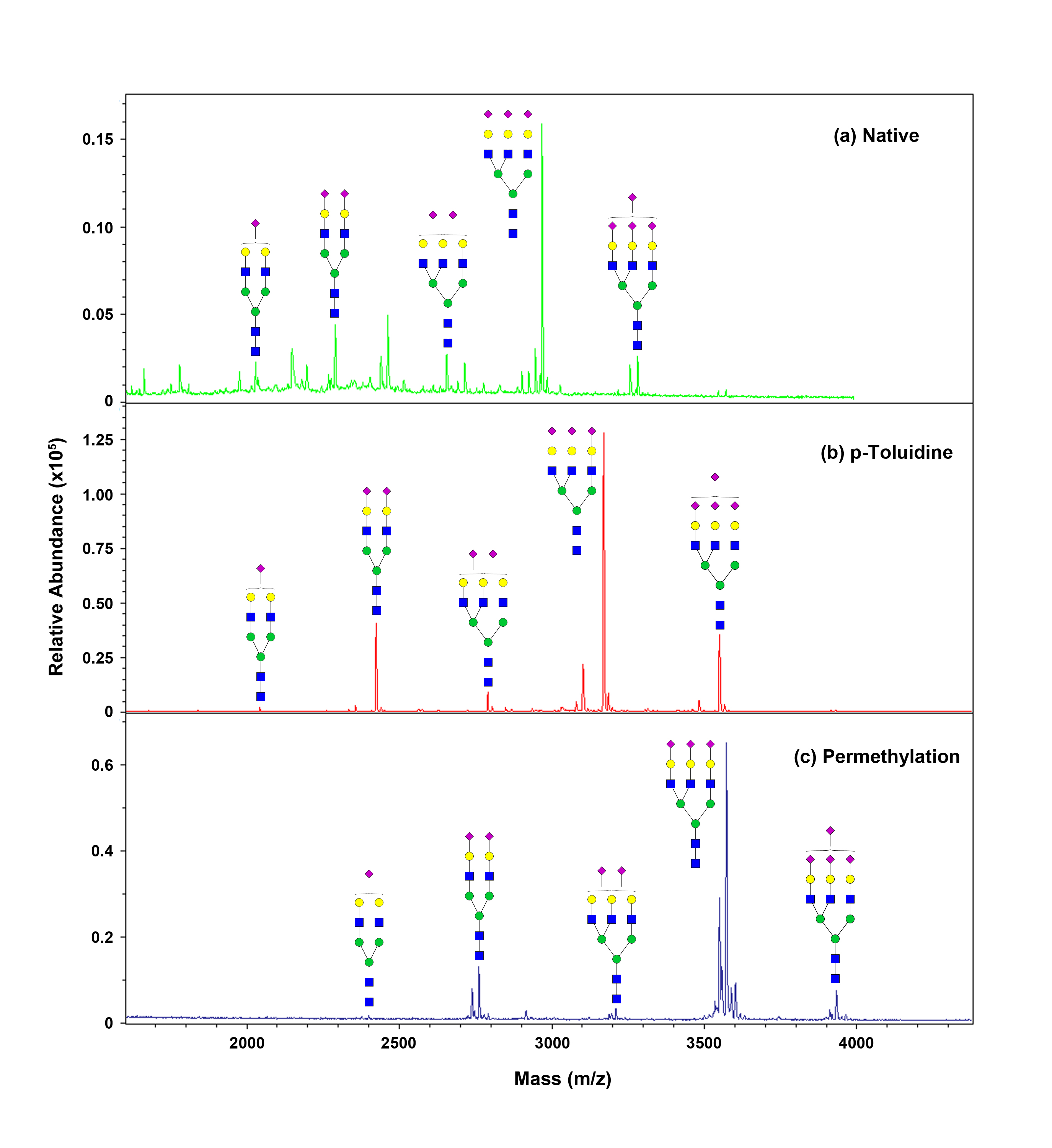
**
